# Do hospitalist physicians improve the quality of inpatient care delivery? A systematic review of process, efficiency and outcome measures

**DOI:** 10.1186/1741-7015-9-58

**Published:** 2011-05-18

**Authors:** Heather L White, Richard H Glazier

**Affiliations:** 1 Department of Health Policy, Management and Evaluation, Faculty of Medicine, University of Toronto, 29 Moffatt Lane, Guelph, ON, N1G 5E8, Canada; 2 The Institute for Clinical Evaluative Sciences, 2075 Bayview Avenue, Room G1-06, Toronto, ON, M4N 3M5, Canada

## Abstract

**Background:**

Despite more than a decade of research on hospitalists and their performance, disagreement still exists regarding whether and how hospital-based physicians improve the quality of inpatient care delivery. This systematic review summarizes the findings from 65 comparative evaluations to determine whether hospitalists provide a higher quality of inpatient care relative to traditional inpatient physicians who maintain hospital privileges with concurrent outpatient practices.

**Methods:**

Articles on hospitalist performance published between January 1996 and December 2010 were identified through MEDLINE, Embase, Science Citation Index, CINAHL, NHS Economic Evaluation Database and a hand-search of reference lists, key journals and editorials. Comparative evaluations presenting original, quantitative data on processes, efficiency or clinical outcome measures of care between hospitalists, community-based physicians and traditional academic attending physicians were included (*n *= 65). After proposing a conceptual framework for evaluating inpatient physician performance, major findings on quality are summarized according to their percentage change, direction and statistical significance.

**Results:**

The majority of reviewed articles demonstrated that hospitalists are efficient providers of inpatient care on the basis of reductions in their patients' average length of stay (69%) and total hospital costs (70%); however, the clinical quality of hospitalist care appears to be comparable to that provided by their colleagues. The methodological quality of hospitalist evaluations remains a concern and has not improved over time. Persistent issues include insufficient reporting of source or sample populations (*n *= 30), patients lost to follow-up (*n *= 42) and estimates of effect or random variability (*n *= 35); inappropriate use of statistical tests (*n *= 55); and failure to adjust for established confounders (*n *= 37).

**Conclusions:**

Future research should include an expanded focus on the specific structures of care that differentiate hospitalists from other inpatient physician groups as well as the development of better conceptual and statistical models that identify and measure underlying mechanisms driving provider-outcome associations in quality.

## Background

In recent years, escalating healthcare costs, a rising prevalence of chronic comorbid diseases and increasing dependence on new technologies have combined to change the nature of inpatient care in North America. Faced with a growing need for cost-effective delivery, hospitals increasingly require that their practicing physicians enhance patient flow and lower operating costs while improving the clinical quality of care provided to their patients. In light of these demands, many hospitals have adopted the hospitalist model as one of the primary methods of achieving these objectives. First introduced in 1996, hospitalists, defined as physicians who specialize in delivering comprehensive medical care to hospitalized patients, have become one of the dominant groups of healthcare providers of inpatient care in North American hospitals [1]. Under the hospitalist model, unattached patients and patients whose primary care physicians do not provide inpatient services are transferred to the care of a hospitalist upon admission to a given institution. Acting as the case manager, the hospitalist's role is to coordinate and integrate care for their assigned patients, which includes generating and reviewing clinical data; making decisions regarding necessary tests, treatments and procedures; and facilitating access to subspecialty and postacute services [1,2]. Upon discharge, patients are returned to the community under the care of their primary care physicians (if they have one), while the hospitalist goes on to care for the next hospital admission. This defining characteristic differentiates hospitalists from their colleagues. Historically, inpatient physicians managed the day-to-day care of their hospitalized patients while maintaining active outpatient practices in either an office or a clinic-based setting. This provided both physicians and patients with some continuity of care, allowing for the development of relationships and medical histories between patient and provider. In contrast, the hospitalist movement represents a shift toward generalized hospital-based care whereby hospitalists provide attention to all routine medical needs throughout the course of hospitalization, but maintain minimal responsibility for outpatient or follow-up care once a patient is discharged [3,4].

Advocates of the hospitalist model argue that hospitalists offer a number of advantages compared with traditional inpatient physician models. The on-site availability of a hospitalist ensures that a dedicated provider is readily available to answer questions, order and manage tests and respond during acute medical crises. By specializing in the management and treatment of common inpatient conditions, this routinization of care is also argued to enhance hospitalists' clinical expertise in complex and comorbid disease management, translating to improved clinical processes and potentially better patient outcomes in comparison to their colleagues, who may manage fewer cases of a given condition over the same period of time [5-7]. On-site availability could also condense the timing of treatments and consultations, thus increasing the efficiency of discharge planning and allowing the hospitalist more time to communicate with patients, their families and patients' primary care providers [8]. At the same time, the hospitalist model represents the purposeful introduction of discontinuity in care. Patients are transferred between providers at admission, discharge and throughout the course of hospitalization. Each transition increases the risk for medical errors and adverse events and jeopardizes both the continuity and quality of care [7,9]. Since the hospitalist enters with no firsthand knowledge of a patient's medical history, he or she may be inclined toward more aggressive, technology-based care, which could translate to the use of more diagnostic tests and higher costs to establish the baseline health status of the patient. Hospitalists may not always be aware of a patient's and family's wishes regarding resuscitation or rescue measures [9], and while each transfer of care provides an opportunity for improved communication between providers, delayed communication or inaccuracies in information transfer may have substantial implications for outpatient follow-up, patient safety, provider satisfaction and overall system utilization [10-12].

In 1998, researchers began evaluating the performance of newly instituted hospitalist programs by comparing full-time hospitalists to traditional academic attending physicians or community-based physicians on the basis of core indicators of effectiveness and efficiency [13,14]. While multiple comparative studies of hospitalists' performance have been published since 1998, substantial disagreement still exists regarding whether and how hospitalists improve the quality of inpatient care delivery. While previous reviews have suggested that hospitalists can lower operating costs and reduce the average length of stay without adversely affecting clinical outcomes [15-17], the validity of findings continues to be scrutinized as a result of inconsistent and vague definitions of hospitalist interventions, poor study designs and inadequate risk adjustment [18-20].

The current systematic review synthesizes the findings of 65 evaluations of hospitalist performance to determine whether the hospitalist model improves the quality of inpatient care delivery compared to traditional inpatient physician models. After proposing a conceptual framework for evaluating hospitalist performance, major findings are summarized according to three core areas of quality: the processes of care delivery, operating efficiency and clinical outcomes of treatment. We also critique the methodological quality of selected publications, exploring whether the quality of hospitalist evaluations have improved over time and offering recommendations to guide the design and analysis of future comparative evaluations.

### New contribution

Although several systematic reviews of hospitalist care have been published, most recent reviews were restricted to specific subsets of the hospitalist literature (high-quality articles using adult inpatient populations [15], communication and information transfer at discharge between hospital-based and primary care physicians [21] and paediatric hospitalists [17]), warranting an updated, in-depth synthesis of the larger body of evidence on overall hospitalist performance. A comprehensive, systematic review incorporating all hospitalist practice styles and inpatient populations was published by Coffman and Rundall [19]. Since the publication of that 2005 review, the number of peer-reviewed comparative studies of hospitalists' quality has tripled. These recent evaluations are important additions to the literature, as they include an expanded focus on the processes of care delivery and on the performance of hospitalists relative to primary care physicians who choose to maintain hospital privileges, both of which improve the generalizability of new evidence for the growing sector of nonacademic hospitals interested in implementing and evaluating hospitalist programs. In addition, many hospitalists have begun broadening their clinical roles, providing newborn or paediatric care; medical comanagement of surgical, cardiac, psychiatric and intensive care unit (ICU) patients; and long-term palliative care [22-25]. This review includes the addition of 12 never-reviewed studies focused solely on these areas of role diversity, many of whose findings deviate from the performance trends seen among hospitalists in a general medical service. This review also includes the first formal methodological critique of the literature, highlighting reporting and analytic concerns which persist and threaten the internal and external validity of reported findings. Finally, we propose a novel conceptual framework for evaluating and synthesizing hospitalist performance on the basis of Donabedian's [26] structure-process-outcome framework for assessing quality in healthcare settings. By situating the empirical findings within an underlying framework, we are able to clarify which structural characteristics of physicians' practices may drive variations in provider performance, which in turn can aid future researchers in organizing and controlling for potential determinants of quality.

### Conceptual framework

In 1966, Donabedian [26] proposed a three-concept framework for analyzing quality improvement wherein the organizational structures of healthcare settings interact with the processes of care delivery to influence clinical, interpersonal and organizational outcomes. According to Donabedian [26], structural indicators of quality refer to the professional, institutional and organizational resources and policies associated with the provision of care and include staffing models, training, credentials and facility resources. Process indicators refer to the things done to and for the patient by providers during the healthcare encounter [27] and can be categorized into two broad types: (1) clinical processes, which include the types of services delivered as well as the appropriateness and timeliness of those services, and (2) interpersonal processes. which include patient-provider and provider-provider communications, patient education and the cultural sensitivity of care [27]. Finally, outcome indicators of quality refer to the end states resulting from care, which may include changes in patient morbidity, mortality, resource utilization, satisfaction and overall quality of life [27]. Donabedian [26,28] noted that these three categories are not independent, but linked in an underlying framework whereby good organizational structures should promote good processes, and good processes in turn should drive better outcomes. It is important to note that while the presence of either structures or processes alone can enable the provision of quality healthcare, they cannot in isolation ensure it [29].

**Figure 1:**
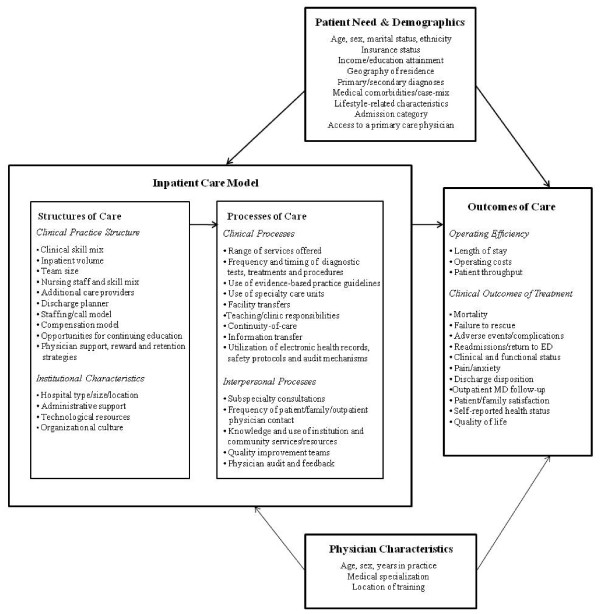
**Conceptual framework for evaluating hospitalist performance in integrating structures, processes, and outcomes of care**.

We propose the conceptual framework illustrated in Figure 1 as a map for understanding, evaluating and synthesizing the quality of hospitalist care while accounting for differences in program designs, institutional resources, provider characteristics and clinical risk. Within the hospitalist literature, the physicians' clinical practice structures represent the key comparative measure of interest, along with institutional characteristics, resources and policies that support the provision of care. While the specific structure of hospitalist programs vary across institutions, common components that distinguish hospitalists from their colleagues include their enhanced expertise and experience in managing common inpatient conditions, greater in-hospital availability and higher volume of inpatient care delivered. Equally important but not often explored factors include nursing staff to patient ratios, administrative resources and organizational cultures that support hospitalist hiring and retention. Process measures reflecting the quality of hospitalist care may include the frequency and timing of diagnostic tests; treatments, procedures and consultations; adherence to evidence-based clinical practice guidelines; utilization of safety protocols, error detection mechanisms and use of electronic medical records; regularity of patient, family and outpatient physician consultations; and opportunities for physician audit and feedback. Finally, outcome measures of quality can reflect both the efficiency of care delivery (for example, length of stay, hospital costs, emergency department processing time) as well as clinical outcomes of treatment (for example, mortality rates, patients' pain and functional status, and patient and family satisfaction). Posthospital outcomes, such as readmission rates, returns to the emergency department and continuity of care/follow-up, can also be examined. Recognizing that patient assignment to providers and subsequent health outcomes are rarely influenced by structural and process inputs alone, we expand on Donabedian's [26] framework to include patients' need for care, patients' basic demographics and the characteristics of physicians involved in the care process.

## Methods

### Search strategy

A comprehensive search of the literature was conducted using MEDLINE, Embase, Science Citation Index, CINAHL and the NHS Economic Evaluation Database for the following exploded medical subject heading terms and keywords: "hospitalist" and "hospital-based medicine." The search was restricted to abstracts published between January 1996 and December 2010, excluding conference abstracts. No language restrictions were imposed. Additional citations were then identified through manual searches of the references and works-cited lists of selected articles as well as previous systematic reviews, relevant journals (*Journal of Hospital Medicine *and *Journal of General Internal Medicine*) and key editorials.

### Article selection

**Figure 2:**
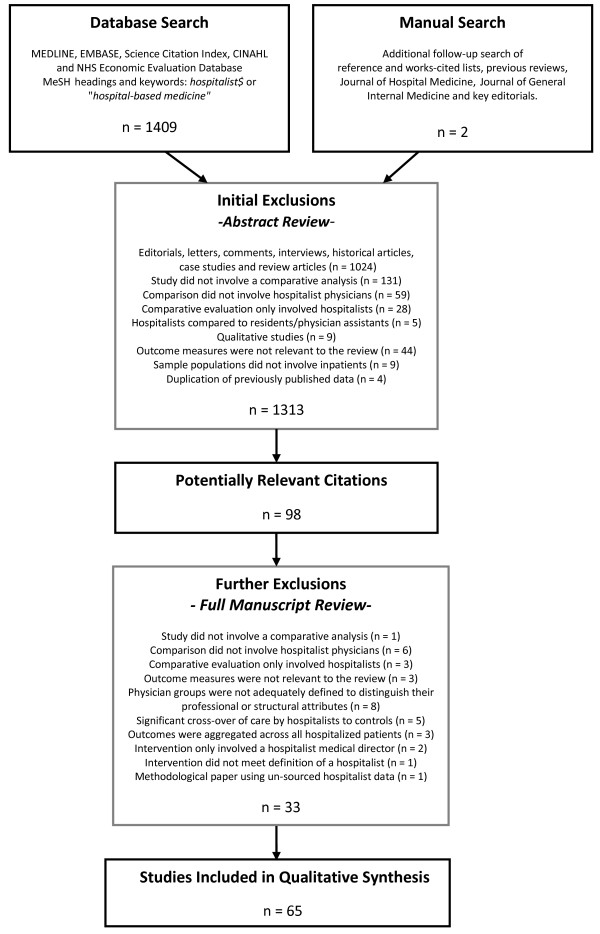
**Flow of information throughout the article selection process used in this review**.

The above-described strategy identified 1,411 electronic citations for which the abstracts were subsequently retrieved and screened by at least one author. Selection criteria for inclusion were as follows: Eligible articles had to (1) describe a comparative analysis between physicians identified or labelled as 'hospitalists' and traditional inpatient physician models involving community-based physicians, traditional academic attending physicians or a combination of both; (2) generate original, quantitative data in one of the three healthcare quality areas of interest (that is, processes of care, operating efficiency and/or clinical outcomes of treatment); (3) differentiate hospitalists from their counterparts in terms of their structural attributes (that is, time spent on-site, patient volume, clinical skill mix); and (4) include a sample population of hospitalized patients. Using these prespecified criteria, abstracts were independently assessed, with any discrepancies resolved by consensus. Seventy-seven articles met these initial inclusion criteria. Upon examination of the full papers, five of these articles were excluded because control patients received significant cross-over of care from the hospitalist physicians [30-34], and three other articles were excluded because healthcare quality was examined among hospitals with and without hospitalists, regardless of whether the sampled patients actually received direct hospitalist care [35-37]. Two papers were excluded because the intervention involved the addition of a hospitalist medical director, as opposed to a hospitalist physician, providing direct inpatient care [38,39], and one paper was excluded because the intervention did not meet a widely accepted definition of a hospitalist program in that no physician spent more than 25% of his or her professional time working as an inpatient specialist [40]. Finally, one methodological paper was excluded because unsourced data on hospitalist performance were used to illustrate the application of a risk adjustment strategy [41]. This left 65 comparative evaluations that were included in our review. The flow of information throughout the selection process is shown in Figure 2.

### Data extraction

One author (HLW) extracted data on each study's design, sample and source population characteristics, institutional setting, a description of hospitalist and comparative care models, risk adjustment techniques employed, and relevant findings. Hospitalist practice models were then classified into three broad design types: private hospitalists hired on contract to provide inpatient care at one or more institutions, salaried faculty hospitalists with no teaching responsibilities, and academic hospitalist attending physicians who worked on the inpatient unit for three to twelve months per year and were involved in the training of residents and medical students. Comparison physicians were similarly classified according to the following traditional practice models: outpatient practices (general practitioners/family physicians, general internists, and paediatricians) and traditional academic attending physicians who served on the inpatient unit for one to three months per year supervising residents and medical students and maintaining outpatient clinic hours while on-service. Multiple practice types and the use of comanagement models, physician's assistants, nurse practitioners, and discharge planners are indicated where appropriate.

### Synthesis of evidence

The major findings from the included studies were synthesized within our conceptual framework according to the following three areas of quality: processes of care delivery, operating efficiency, and clinical outcomes of treatment. Relationships are summarized by each indicator's percentage change, direction, and statistical significance. A summary of the 65 included articles and their overall findings are presented in Table 1, while detailed results from individual analyses can be found in Additional file 1: Individual study results on hospitalist performance. Where available, the results of the authors' risk-adjusted models are presented and are considered significant when a *P *value ≤0.05 was reported. Summary measures based on unadjusted analyses are indicated by asterisks (*), and those without accompanying *P *values or confidence intervals are indicated by an alveolar click (ǂ).

**Table 1:** Summary of articles evaluating hospitalist performance (*n *= 65)^a^

							Hospitalist performance		
Source	Design	Hospital type	Study population	Sample	Comparison	Quality score	Processes of care	Operating efficiency	Patient outcomes
Abenhaim *et al. *[44]	RC	Teaching	Adults admitted to either GMS or medical short-stay unit	2,722	F vs. TWS	8	↑,*,ǂ	↑,*,ǂ	↑,*,ǂ
Auerbach *et al. *[85]	RC	Teaching	Adults admitted to GMS	5,308	A vs. C	24	-	↑	↑
Auerbach and Pantilat [76]	RC	Teaching	Adults admitted to GMS who died while in hospital	148	A vs. C	21	↑		↑
Batsis *et al. *[94]	B/A	Teaching	Seniors admitted for surgical repair of hip fracture	466	F^b ^vs. TWS	13			-,*
Bekmezian *et al. *[79]	RC	Paediatric teaching	Paediatric patients with oncologic, hematologic or gastroenterologic disease	925	F vs. TWS	17		↑	↓,*
Bell *et al. *[52]	QE	Teaching (six sites)	All patients admitted to GMS	1,078	Mixed practice types	6	-,*		
Bellet and Whitaker [80]	B/A	Paediatric teaching	Paediatric patients admitted to GMS	1,440	A vs. TWS and C	24	-,*	↑	↓,*
Boyd *et al. *[95]	RC	Teaching	Paediatric patients admitted to GMS	1,009	P vs. TWS	16		↓	
Carek *et al. *[96]	RC	Community	Adults admitted to GMS	5,453	P vs. C P vs. TWS	21		↓,* ↓	-,* -
Craig *et al. *[61]	RC	Community (16 sites)	Adults admitted from one HMO to GMS		P vs. C	8		↑,ǂ	-,ǂ
Davis *et al. *[65]	RC	Community	All patients admitted to GMS	2,124	P^c ^vs. C	13	↑,*	↑	-
Dhuper and Choksi [86]	B/A	Teaching	All patients admitted to GMS	10,966	A^c ^vs. TWS	14			↑,*
Diamond *et al. *[13]	B/A	Teaching	Adults admitted to GMS	3,299	A vs. C	16		↑,*	↑,*
Dwight *et al. *[45]	RC	Paediatric teaching	Paediatric patients admitted to GMS	3,807	F vs. TWS	22	-	↑	-
Dynan *et al. *[97]	RC	Teaching	All patients admitted to GMS	5,543	F^c ^vs. TWS	14		↑	-
Everett *et al. *[98]	RC	Community	All patients admitted to GMS	11,750	P vs. C	15		↑	-
Everett *et al. *[87]	RC	Teaching	All patients admitted to GMS	22,792	P vs. C P vs. TWS	14		↑ ↓	- ↑
Freese *et al. *[60]	B/A	Community	All patients admitted to GMS		P vs. C	6	↑,*,ǂ	↑,*,ǂ	
Gittell *et al. *[63]	RC	Community	All patients admitted to GMS	6,686	P vs. C	7		↑	↑
Go *et al. *[51]	QE	Teaching (six sites)	Adults admitted to GMS with diagnosis of acute upper gastrointestinal haemorrhage	450	A vs. TWS	22	-,*	↓	-
Gregory *et al. *[99]	B/A	Teaching	All patients admitted to GMS	402	F vs. TWS	8		↑,*	-,*
Hackner *et al. *[68]	PC	Teaching	Adults on Medicaid admitted to GMS	1,637	A vs. C	19	↑,*	↑,*	-,*
Halasyamani *et al. *[100]	RC	Teaching	Adults admitted to GMS	10,595	P vs. C A vs. C	21		↑ ↑	- -
Huddleston *et al. *[48]	RCT	Teaching	Adults undergoing elective hip or knee arthroplasty	469	F^b ^vs. TWS	26		↑	↑
Kaboli *et al. *[50]	QE	Teaching	All patients admitted to GMS	1,706	A vs. TWS	23		↑	-
Kearns *et al. *[56]	QE	Teaching	All patients admitted to GMS	4,455	A vs. TWS	26	-,*	-	-
Khasgiwali *et al. *[101]	RC	Teaching	All patients admitted to GMS	1,916	P and A vs. TWS	14	-,*	-,*	-,*
Krantz *et al. *[58]	B/A	Teaching	All patients admitted to chest pain observational unit	493	P^b ^vs. TWS	19		↑,*	-,*
Kulaga *et al. *[78]	RC	Teaching	All patients admitted to GMS	2,707	A vs. C	8		↑,*,ǂ	↑,*
Kuo *et al. *[102]	RC	Mixed (4,359 sites)	5% national sample of admissions among Medicare beneficiaries	314,590	Mixed practice types	16		↑	
Landrigan *et al. *[103]	TS	Paediatric teaching	Paediatric patients admitted to GMS from three HMOs	7,748	A^c ^vs. C	15		↑	-
Lindenauer *et al. *[74]	RC	Teaching	Adults admitted with heart failure	326	P and A vs. C	14	↑	↑	-,*
Lindenauer *et al. *[92]	RC	Mixed (45 sites)	Adults admitted with pneumonia, heart failure, chest pain, stroke, UTI, COPD or acute MI	76,926	Mixed practice types	20		↑	-
Maa *et al. *[62]	B/A	Teaching	Adults undergoing surgical appendectomy		A vs. TWS	7		↑,*	
Meltzer *et al. *[54]	QE	Teaching	All patients admitted to GMS	6,511	A vs. TWS	20		↑	↑
Molinari and Short [104]	B/A	Community	Adults admitted from one HMO	1,319	P^c ^vs. C	8		↑	
Ogershok *et al. *[66]	B/A	Paediatric teaching	Paediatric patients admitted to GMS	2,177	A vs. TWS	14	↑,*	↑,*	-,*
Palacio *et al. *[88]	RC	Teaching	All patients admitted to GMS	5,943	F vs. TWS	11		↑,*	↑
Palmer *et al. *[49]	QE	Teaching	All patients admitted to GMS	2,464	A^c ^vs. TWS	25	↑,*	↑	↑
Parekh *et al. *[105]	RC	Teaching	All patients admitted to GMS	2,552	A vs. TWS	19		-	-
Phy *et al. *[82]	B/A	Teaching	Older adults admitted for surgical repair of hip fracture	466	F^b,c ^vs. TWS	15		↑	-,*
Pinzuer *et al. *[77]	B/A	Teaching	Adults admitted for lower-extremity salvage or reconstructive surgery	140	F^b ^vs. TWS	9		↑	↓,ǂ,*
Ravikumar *et al. *[83]	B/A	Teaching (four sites)	Adult surgical patients	39,769	F^b,c ^vs. TWS	8		↓,ǂ,*	↓,*
Reddy *et al. *[72]	RC	Teaching	All patients admitted with community-acquired pneumonia	151	A vs. C and TWS	9	-	-	
Rifkin *et al. *[70]	RC	Community	Adults admitted with community-acquired pneumonia	455	P vs. C	20	↑,*	↑	-,*
Rifkin *et al. *[106]	RC	Teaching	All patients admitted to GMS	11,388	F vs. C	18		-	
Rifkin *et al. *[69]	RC	Community	All patients admitted with community-acquired pneumonia	158	F vs. C	11	↑,*		
Roy *et al. *[30]	RC	Teaching	Adults admitted with hip fracture	118	F vs. C	9		↑,*	
Roytman *et al. *[67]	RC	Teaching	Adults admitted with congestive heart failure	342	F vs. C	20	↑	↑	↑
Salottolo *et al. *[89]	B/A	Teaching	Adult trauma admissions	500	F vs. TWS	5		↓	-
Scheurer *et al. *[107]	RC	Mixed (29 sites)	All patients admitted with bacterial pneumonia	11,969	Mixed practice types	7		↑,*	
Schneider *et al. *[53]	QE	Teaching (six sites)	All admissions to GMS with HIV infection	1,207	A vs. TWS	17	-	-	-
Sharma *et al. *[9]	RC	Mixed (11 sites)	Older adults on Medicaid with advanced lung cancer	21,183	Mixed practice types	14	↓		
Simon *et al. *[84]	B/A	Paediatric teaching	Paediatric patients undergoing spinal fusion	759	F^b ^vs. TWS	8		↑	
Sloan *et al. *[24]	B/A	Community VA	Adults admitted to inpatient psychiatric unit	1,409	F^c ^vs. C^c^	18		-,*	↑,*
Smith *et al. *[71]	RC	Teaching	Adults admitted with community-acquired pneumonia	45	P vs. C	14	-	↓	-,*
Somekh *et al. *[59]	RC	Teaching	Admissions to GMS or cardiac observational unit for chest pain	750	F vs. C F vs. cardiologist	11	↑,* ↓,*	↓,ǂ ↓	- ↓
Southern *et al. *[108]	RC	Teaching	All patients admitted to GMS	9,037	A vs. TWS	19		↑	-
Srivastava *et al. *[64]	B/A	Paediatric teaching	Paediatric patients from one HMO admitted with asthma, dehydration or viral illness	1,970	A vs. TWS	19		↑	
Stein *et al. *[73]	RC	Teaching	Adult admitted with community-acquired pneumonia	237	A vs. C	11	-,*	↑,*	-,*
Tenner *et al. *[57]	B/A	Paediatric teaching (two sites)	Paediatric admissions to ICU	1,211	P vs. TWS	17		↑	↑
Tingle and Lambert [109]	RC	Teaching	Adults admitted to GMS	529	F vs. TWS	14		-	-,*
Vasilevskis *et al. *[75]	RC	Teaching (six sites)	Adults with heart failure admitted to GMS	372	Mixed practice types	18	-	-	↑
Wachter *et al. *[55]	QE	Teaching	All patients admitted to GMS	1,623	A vs. TWS	18	-,*	↑	-
Wells *et al. *[110]	PC	Community	Paediatric patients admitted to GMS	181	P vs. C	5		↑	-,*

^a^RCT, randomized, controlled trial; QE, quasi-experimental design; TS, time series; PC, prospective cohort; RC, retrospective cohort; B/A, before versus after; CS, cross-sectional survey; GMS, general medical service; HMO, health maintenance organization; UTI, urinary tract infection; COPD, chronic obstructive pulmonary disease; MI, myocardial infarction; HIV, human immunodeficiency virus; ICU, intensive care unit; P, private hospitalist attending physician; F, nonacademic faculty hospitalist attending physician; A, academic hospitalist attending physician; C, community-based physician; TWS, traditional academic attending physicians with teaching responsibilities; ^b^hospitalists were comanaging their patients' care with comparison healthcare providers; ^c^use of physician's assistants, nurse practitioners and/or discharge planners in the provision of care; ↑ indicates improved performance by hospitalists; - indicates no difference in performance between providers; ↓ indicates worse performance by hospitalists; ǂ indicates that a *P *value or confidence interval was not provided, so results may or may not be statistically significant; *indicates that results are unadjusted.

To assess the methodological quality of the included literature, we used a 27-item checklist developed by Downs and Black [42] that was designed and validated to gauge the following four areas of methodological quality in both randomized and nonrandomized studies of healthcare interventions: disclosure and/or reporting, internal validity, external validity, and study power. To capture methodological issues specific to reporting within hospitalist interventions, we added five additional questions to the original 27-item checklist regarding the authors' disclosure of (1) funding sources, (2) location of the intervention, (3) whether hospitalists were used exclusively for managing the care of specific inpatient populations, (4) whether incentives (monetary or otherwise) were provided for physicians to enhance their performance, and (5) the role of additional providers in the provision of inpatient care. We added one additional question regarding whether the authors included a power assessment in their article, and one question was excluded on the blinding of participants to intervention allocation because patients are generally aware of who is managing their day-to-day care. To score the methodological quality of each article, a score of 1 was assigned for each of the 32 questions in the checklist answered 'yes' and a score of 0 for each question answered either 'no' or 'unable to determine'. Marks were then summed to provide a total quality score (maximum = 32). The modified checklist and evaluation criteria used to assign all quality ratings are given in Additional file 2: Checklist for assessing study quality, modified from Downs and Black [42]. The systematic review was performed according to the 2009 Preferred Reporting Items for Systematic Reviews and Meta-Analyses (PRISMA) statement [43] (see Additional file 3: PRISMA Checklist: Do Hospitalist Physicians Improve the Quality of Inpatient Care?).

## Results

### Study characteristics

Descriptive characteristics summarizing the 65 articles are presented in Table 2. Sixty-three of the evaluations were conducted in the United States, and the remaining two studies utilized data from Canadian institutions [44,45]. After we screened the hospitalist literature for inclusion in our review, hospitalist programs were adopted in several countries outside North America, including Australia, New Zealand, Argentina, Brazil, Chile, Columbia, Spain, Sweden, and Singapore [18,46,47]. While several editorials and descriptive papers have been published on programs within these countries, no comparative analyses conducted in these countries have appeared in the literature to date.

**Table 2:** Descriptive characteristics of 65 comparative evaluations of hospitalist performance^a^

Study characteristics	Studies, *n *(%)^b^
Country of research	
Canada	2 (3.0)
United States	63 (97.0)
Research design	
Randomized or quasi-randomized controlled trial	9 (13.8)
Interrupted time series	1 (1.5)
Prospective cohort	2 (3.1)
Retrospective cohort	35 (53.8)
Before and after	18 (27.7)
Patient eligibility	
Adult patients only	25 (38.5)
Paediatric patients only	10 (15.4)
Older adult patients only (age ≥65 years)	3 (4.6)
Medicare/Medicaid enrolment	3 (4.6)
HMO/VA enrolment	5 (7.7)
Diagnostic/disease eligibility	
Asthma/bronchiolitis	3 (4.6)
Chest pain	6 (9.2)
Cancer/haematology	2 (3.1)
Chronic obstructive pulmonary disease	4 (6.2)
Community-acquired or bacterial pneumonia	14 (21.5)
Gastrointestinal/digestive disorders	8 (12.3)
Heart failure	9 (13.8)
Human immunodeficiency virus	1 (1.5)
Hypovolemia/dehydration	2 (3.1)
Myocardial infarction	2 (3.1)
Nutritional/metabolic disorders	4 (6.2)
Orthopaedic and other surgical procedures	9 (13.8)
Psychiatric illness/substance dependency	2 (3.1)
Stroke	4 (6.2)
Trauma	2 (3.1)
Urinary tract infection	4 (6.2)
Viral illness	2 (3.1)
Hospital type	
Teaching hospital	54 (83.1)
Community/rural hospital	11 (16.9)
Location of care	
General medical/surgical service	60 (92.3)
Chest pain observation unit	2 (3.1)
Intensive care unit	1 (1.5)
Medical short-stay observation unit	1 (1.5)
Psychiatric unit	1 (1.5)
Hospitalist practice structure^b^	
Private hospitalists	22 (33.8)
Nonacademic faculty hospitalists	26 (40.0)
Academic hospitalist attending physicians	33 (47.7)
Mix of practice structures	
Comparative practice structure^b^	
Community-based physicians	34 (52.3)
Traditional academic attending physicians	41 (63.1)

^a^HMO, health maintenance organization; VA, Veterans Affairs; ^b^percentages may not sum to 100 due to rounding; ^c^number of articles may not sum to 65 as several studies compared more than one physician structure.

Only one of the sixty-five reviewed articles employed a true randomized, controlled study design in which the first patient enrolled was randomly allocated to either hospitalist or traditional care at the time of admission [48]. Subsequent patients were then assigned using concealed, dynamic allocation. Eight additional articles used quasi-randomized designs based on natural experiments in which patients were assigned to either hospitalist or comparative care according to their position in the physicians' call schedules [49-55] or alternating rotations [56]. Randomization appeared to be successful for all but two of these studies [51,52], reporting no statistically significant differences between the intervention and control groups with respect to baseline patient demographics, diagnoses, and underlying comorbidities (*n *= 7; 78%). The remaining 56 evaluations used one of the following observational designs: interrupted time series (*n *= 1; 2%), prospective cohorts (*n *= 2; 3.0%), retrospective cohorts (*n *= 35; 54%), and before and after (*n *= 18; 28%). In most observational studies, the design of the hospitalist intervention precluded randomization as community-based physicians elected to manage their own hospitalized patients.

The majority of studies were not restricted with respect of the ages of study participants (*n *= 27; 42%). Twenty-five evaluations examined outcomes among adults ages 18 and older, three were restricted to older adults (ages 65 and older), and ten focused on paediatric patients. Among the 63 evaluations conducted in the United States, insurance status was rarely used as an exclusion criterion (*n *= 8; 13%). Four studies examined outcomes of hospitalist care among commercial health maintenance organization (HMO) enrolees, three evaluated Medicare or Medicaid recipients, and one involved a source population who received care through Veterans Affairs hospitals [24]. Several evaluations also examined the quality of inpatient care among patients with specific diseases and conditions including orthopaedic, trauma, and other surgical procedures; lung disease, cardiovascular disease, infections, and gastrointestinal disease; metabolic and autoimmune disorders; and mental health issues or substance dependency (see Table 2 for frequencies).

Eighty-three percent of all evaluations were conducted within teaching hospitals or units and involved single-site comparisons (*n *= 54). Of the eleven evaluations conducted across multiple facilities, ten included at least one teaching hospital (91%). While most articles evaluated quality of care among patients in a general medical or surgical service (*n *= 60; 92%), one was restricted to the provision of care within the ICU [57] and one to an inpatient psychiatric unit [24]. One study examined cooperative hospitalist or cardiologist care on a chest pain observation unit designed for patients at low risk for cardiovascular events [58], and one additional article compared hospitalist care on the general medical service to cardiologists working in a similar chest pain unit [59]. Finally, one Canadian study examined performance on a hospitalist-run, short-stay unit in comparison with care provided on a general medical service [44].

Considerable variation exists in the number of study participants and healthcare providers included across evaluations (see Table 3 for summary statistics). The median number of sampled patients was 1,630 (reported in 62 studies), and the median number of hospitalist practitioners was six (reported in 51 studies). In three of the sixty-five evaluations, the overall sample size was not disclosed [60-62], and three additional authors did not report sample sizes within comparison groups [9,63,64]. The number of hospitalists and comparative physicians who provided care to included participants was not reported in 22% (*n *= 14) and 49% (*n *= 32) of publications, respectively. Thirteen evaluations compared the quality of inpatient care among patients managed on academic ward teams led by hospitalist attending physicians with those managed by traditional academic physicians attending on the inpatient service for one to three months per year (20%). Seventeen additional evaluations compared patients in nonteaching hospitalists with those managed by traditional academic attending physicians (26%), and seven compared patients of academic hospitalists to patients managed by community-based physicians (11%). Fourteen evaluations compared the performance of nonteaching hospitalists with community-based physicians (22%), and the fourteen remaining articles involved comparisons across several different physician models (22%). Finally, seven articles examined hospitalist comanagement practices in which hospitalists provided general medical care to patients assigned to surgical (n = 6) or cardiac (n = 1) teaching teams.

**Table 3:** Summary statistics of the 65 comparative evaluations on hospitalist performance

Study characteristics	Value
Study participants (*n *= 62)	
Median	1,630
Mean	10,272.1
Range	45 to 314,590
Hospitalist physicians (*n *= 51)	
Mean	15.4
Median	6
Range	1 to 284
Nonhospitalist physicians (*n *= 37)	
Mean	156.5
Median	46
Range	1 to 1,964
Number of outcomes studied	
Median	4
Mean	4.7
Range	1 to 17
Study quality score (maximum = 32)	
Median	15
Mean	14.9
Range	5 to 26
Significant improvement by hospitalists on ≥1 quality indicator, *n *(%)	
No improvement or worse performance	16 (24.6)
Better quality on ≥1 indicator	46 (70.8)
Unknown/significance not reported	3 (4.6)

### Quality of hospitalist care

Overall, 46 (71%) of the 65 reviewed articles demonstrated improved quality under hospitalist care on at least one indicator. Three additional papers suggested similar trends in performance (4%); however, the authors failed to report the statistical significance of their findings [44,60,61]. Of the remaining nineteen articles, nine (14%) failed to demonstrate any variations in quality between providers, and seven (11%) indicated worse outcomes for patients managed by hospitalists.

#### Process indicators of hospitalist quality

Twenty-six comparative evaluations examined the processes of care delivery between hospitalists and their colleagues. Among these evaluations, twenty-two indicators of clinical processes and five indicators of interpersonal processes were examined. Clinical process indicators included measures of diagnostic and procedural utilization, adherence to evidence-based clinical practice guidelines for the treatment of common conditions and ICU transfers, while interpersonal process indicators explored consultation rates to various subspecialty providers, the frequency of family contact, and communication patterns with patients' primary care physicians. Subspecialty consultation rates were the most commonly explored process indicator of hospitalist quality (*n *= 9; 35%), followed by several indicators of resource utilization, including radiology (*n *= 8; 31%), laboratory testing (*n *= 7; 27%), and the use of haematology services (*n *= 6; 23%). These outcomes were frequently identified retrospectively on the basis of hospital administrative and financial databases, although primary chart abstraction was used for some indicators in 11 evaluations (42%).

On the basis of our review of the literature, there appear to be few differences in the processes of care delivery between hospitalists, traditional academic attending physicians, and community-based physicians. Of the eleven studies conducted to evaluate the utilization of ancillary services (defined as support services other than medical and nursing staff provided to patients in the course of care including diagnostic testing and therapeutic services), only four studies reported significant declines in the number of services used by hospitalists, three of which were based on unadjusted analyses [49,65,66]. None of the authors of these three articles found significant differences in sputum culture or oxygen pressure testing, occupational and/or physical therapy, or dietitian utilization (*n *= 4). Only one of the three articles reported minor improvements in cardiac testing among nonacademic hospitalists compared to community-based physicians; however, the utilization of diagnostic testing by hospitalists remained higher and more invasive than that provided by cardiologists [59].

Only two of nine studies found significant declines (22%) in subspecialty consultation rates [67,68], one of which was based on unadjusted analyses [68]. None of the reviewed articles described improvements in ICU use (*n *= 6), and one article described increased use of ICUs by hospitalists for patients with advanced stage lung cancer during these patients' final hospitalization [9]. While only two comparative studies have looked at communication patterns between inpatient physicians and the patients' primary care providers [52,53], there is no evidence to suggest that hospitalists communicate any better or worse than their colleagues.

Hospitalist and nonhospitalist physicians were equally likely to provide core measures of care for patients with pneumonia and immunosuppression. While Rifkin *et al. *[69] found that hospitalists were more likely to provide deep vein thrombosis prophylaxis and pneumococcal vaccination (or to have documented patients' ineligibility for these treatments), there were no significant differences in door-to-needle time for antibiotic initiation, the appropriateness of antibiotic use, the number of infectious disease or pulmonary consultations, serial chest radiography, ICU use, or smoking cessation counselling in several studies [69-73]. Similarly, in a large multisite trial examining the quality of care provided to inpatients with human immunodeficiency virus (HIV), Schneider *et al. *found no significant differences in processes of care between managing physicians, regardless of the physicians' prior experience in managing patients with known HIV infection [53]. Hospitalists showed no clearer trends in improvement with regard to adherence to evidence-based practice guidelines for cardiac care. While one study reported a slight increase in the assessment of left ventricular ejection fraction among patients with decompensated heart failure [74], another study failed to establish a significant effect [75]. Neither study found differences in angiotensin-converting enzyme inhibitor (ACE-I), angiotensin II receptor blocker (ARB), β-blocker, or warfarin utilization. A third study did report increased use of ACE-I and ARB use by hospitalists within 24 hours of admission; however, hospitalists were also less likely to initiate β-blocker use during hospitalization [67]. No significant differences in cardiac testing, sodium or fluid restriction, or lifestyle counselling were reported in any of these studies.

While less evidence is available regarding best practices for palliative care, the on-site availability of hospitalists may lead to enhanced efforts to communicate with dying patients and their families, resulting in improvements in the quality of end-of-life care. In a comprehensive study of palliative care patterns by academic hospitalists and community-based physicians, Auerbach and Pantilat [76] found that hospitalists were more likely to have documented discussions with patients and/or their families regarding their wishes for care. Although a higher proportion of hospitalist-treated patients were full code at admission, there was a trend toward more hospitalist patients' receiving comfort care at the time of death (*P *= 0.14). Nonhospitalist healthcare providers were similar in their use of opioids, although hospitalists were more likely to prescribe long-acting benzodiazepines in the 48 hours prior to death to aid patients' comfort and anxiety.

#### Hospitalists as efficient providers of inpatient care

**Figure 3:**
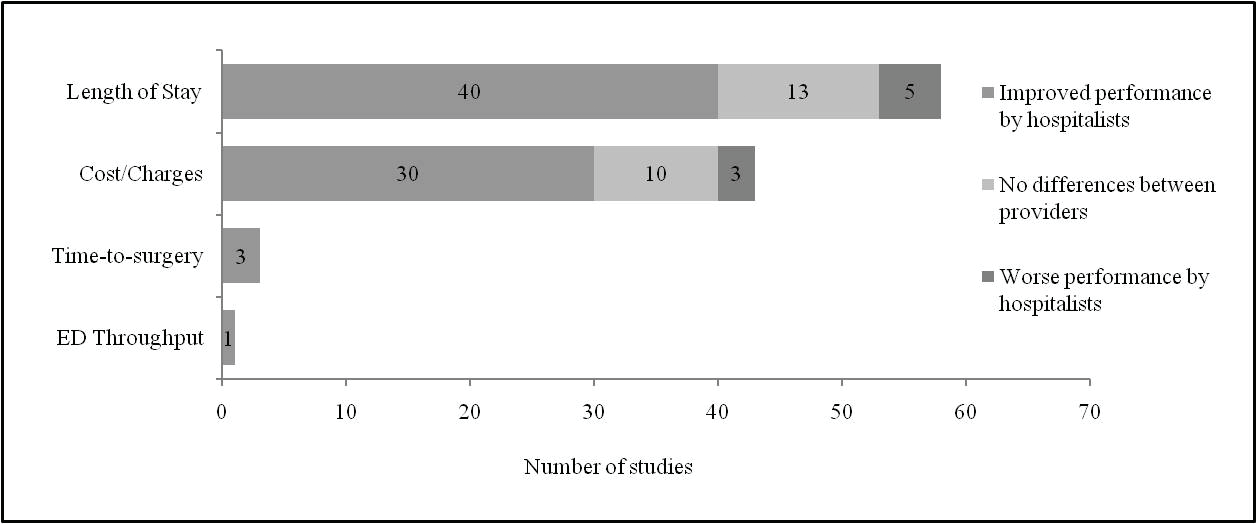
**Summary of findings regarding hospitalist performance and the efficiency of inpatient care**.

Fifty-nine articles examined the efficiency of care delivery between inpatient physician models, the findings of which are summarized in Figure 3. Length of stay and total hospital costs were the two main indicators used to assess the efficiency of hospitalist care, although two additional indicators for emergency department processing and time to surgery were also examined. Outcomes were often identified retrospectively from hospital financial databases.

As illustrated in Figure 3, the majority of reviewed articles suggest that hospitalists can improve the quality of inpatient care delivery by enhancing their hospital's operating efficiency. Thirty-five of the fifty-eight articles that examined average or median length of stay found that patients managed by hospitalists had significantly shorter hospital stays compared to those who received traditional models of inpatient care (60%). Five additional papers suggested similar declines (9%); however, the authors failed to disclose the statistical significance of their findings [44,60,61,77,78]. Shorter lengths of hospital stays persisted across all hospitalist practice models: Twelve (80%) of fifteen articles comparing nonacademic hospitalists to community-based physicians and eleven (61%) of eighteen articles comparing nonacademic hospitalists with traditional academic attending physicians showed shorter patient hospital stays under hospitalist care. Eighty-eight percent of studies demonstrated shorter lengths of stay among patients treated by academic hospitalists compared to those treated by community-based physicians (seven of eight studies), and the figure was sixty-two percent among academic hospitalists compared to traditional academic attending physicians (eight of thirteen studies). Only 55% of evaluations demonstrating shorter lengths of stay reported adjusted measures of effect estimated on the basis of various regression models (*n *= 22), and less than one-third of these used methods to adjust for the clustering of patients within physicians (*n *= 6). Thirteen evaluations found no significant differences in length of stay between healthcare providers (22%), the majority of which involved comparisons between hospitalist and traditional attending physicians (*n *= 9, 69%), and seven evaluations reported longer lengths of stay among hospitalists (12%). Fifty-seven percent of these evaluations involved comparisons between private hospitalists hired on contract and traditional academic attending physicians (*n *= 4).

Of the 43 articles examining hospital costs or charges, 27 showed significant reductions in the average or median cost of care under the hospitalist model (63%). Three additional papers suggested similar cost savings; however, the authors of these papers failed to disclose the statistical significance of their findings [60,61,78]. Cost reductions were reported in eight (67%) of twelve articles among nonacademic hospitalists compared to community-based physicians and in four (44%) of nine articles among nonacademic hospitalists compared to traditional academic attending physicians. All studies showed lower costs of care for patients treated by academic hospitalists compared to those treated by community-based physicians (*n *= 7), and 63% of investigations showed similar cost reductions between nonacademic hospitalists and traditional academic attending physicians (seven of eleven studies). Three evaluations reporting lower costs by hospitalists added length of stay as a covariate to their regression analyses [55,79,80]. In doing so, cost savings were no longer significant, suggesting that reductions in cost are likely the result of shorter length of stay as opposed to a reduction in the type and intensity of services provided, a finding supported by our previous analysis of process indicators which showed no reductions in the utilization of ancillary services by hospitalists.

Hospitalists may also improve the timeliness of emergency surgical care. In three studies where admission and preoperative assessments were conducted by hospitalists as opposed to a member of the surgical team, mean time to surgery was reduced by 35% to 50% [62,81,82]. Along with improvements in efficiency prior to surgery, overall lengths of stay for surgical patients comanaged by hospitalists were reduced in all studies [48,77,82-84], although none demonstrated associated reductions in costs [48,77]. Last, while hospitalist teams are often argued to improve emergency department flow through active and ongoing bed management, only one evaluation to date has reported significant improvements in emergency department processing [58], but no form of risk adjustment was used in their analyses.

#### Clinical outcomes under hospitalist care

**Figure 4:**
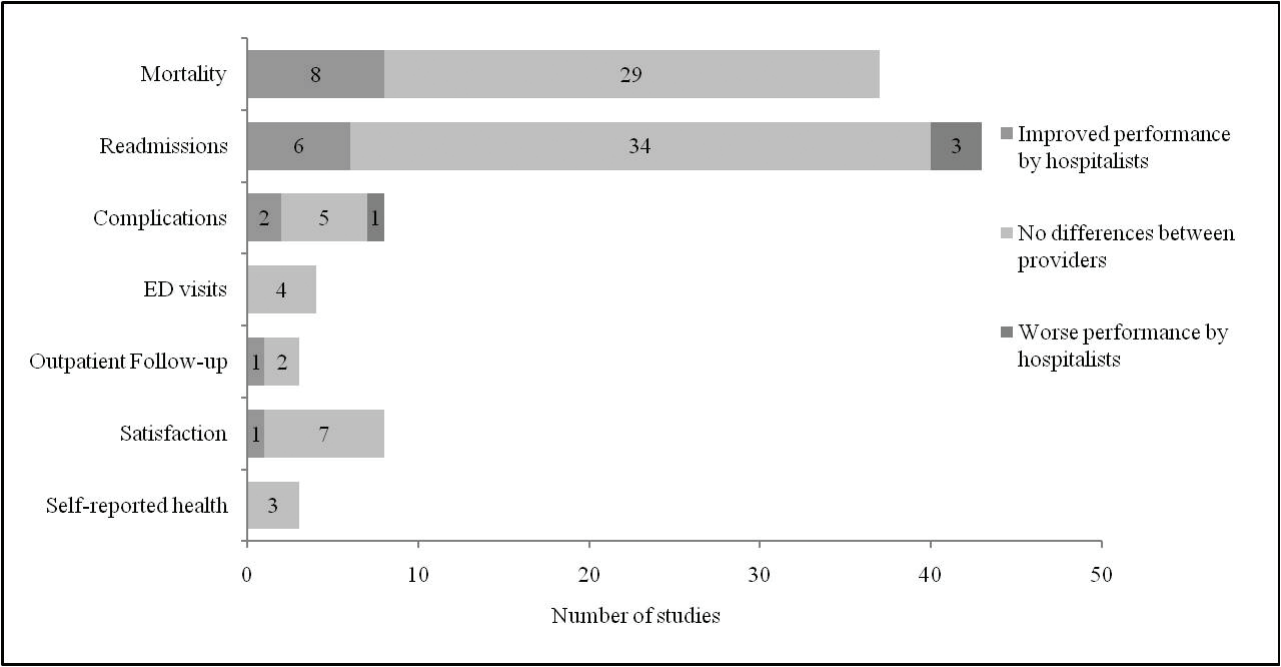
**Summary of findings regarding hospitalist performance and clinical outcomes of treatment**.

Fifty-one evaluations examined the relationship between hospitalist delivery models and clinical outcomes of treatment. Outcomes were frequently identified retrospectively using patient-level data captured in discharge databases and/or death registries (*n *= 35; 69%), and chart validation occurred in five of these evaluations (*n *= 14%). A summary of the findings is displayed in Figure 4.

Although our analysis suggests that hospitalists can improve the efficiency of inpatient care delivery, there is little evidence to suggest this translates into measurable improvements in the effectiveness of care provision to their patients. Thirty-seven studies analyzed mortality or survival rate as one indicator of hospitalist quality. Mortality was most frequently defined as occurring 'in-hospital' (*n *= 35; 95%), although seven studies looked at death within other periods after discharge (thirty days, sixty days, six months, and one year; 19% overall). Seven of the thirty-five evaluations reported significant declines (20%) in mortality rates among hospitalist providers, including two quasi-experimental studies [49,54] and five observational studies [57,67,83,85,86]. Readmissions, usually to the same facility, were examined in 43 evaluations (within seventy-two hours; seven, ten, fourteen, or thirty days; and one year), with the majority finding no differences between providers (*n *= 34; 79%). Six authors reported declines in readmissions within 30 days of discharge [24,44,58,87,88]; however, only two were from risk-adjusted regression models [87,88] and one author failed to disclose the statistical significance of the relationship [44]. In addition, three studies reported higher readmissions among hospitalists, all of which involved comparisons to traditional academic attending physicians [59,79,80].

Additional outcome indicators included in-hospital complications and adverse events (*n *= 8), emergency department and outpatient follow-up visits within 30 days of discharge (*n *= 4 and *n *= 3, respectively), patient and/or parent satisfaction (*n *= 8), and patients' self-reported health (*n *= 3). Five of the eight articles which examined complications or adverse events found no significant differences between providers [51,67,82,86,89]. Huddleston *et al. *[48] observed a reduction in surgical complications in orthopaedic patients whose postoperative medical care was managed by hospitalists. Abenhaim and colleagues [44] also reported reductions in complications; however, patients in that study were preferentially admitted to hospitalist care based on a shorter anticipated length of hospital stay, and the analyses did not adjust for differences in the severity of patients' conditions as well as in case mix. Finally, a recent study published by Pinzuer *et al. *[77] found that high-risk patients undergoing lower-extremity salvage or reconstructive surgery had higher complication rates when comanaged by hospitalists as compared to prior management by the surgical team alone. No differences were found between care providers on any of the remaining outcomes, including rates of return to emergency department, outpatient follow-up visits to the patient's primary care provider, patient satisfaction, or patient self-reported health.

### Methodological critique

**Figure 5:**
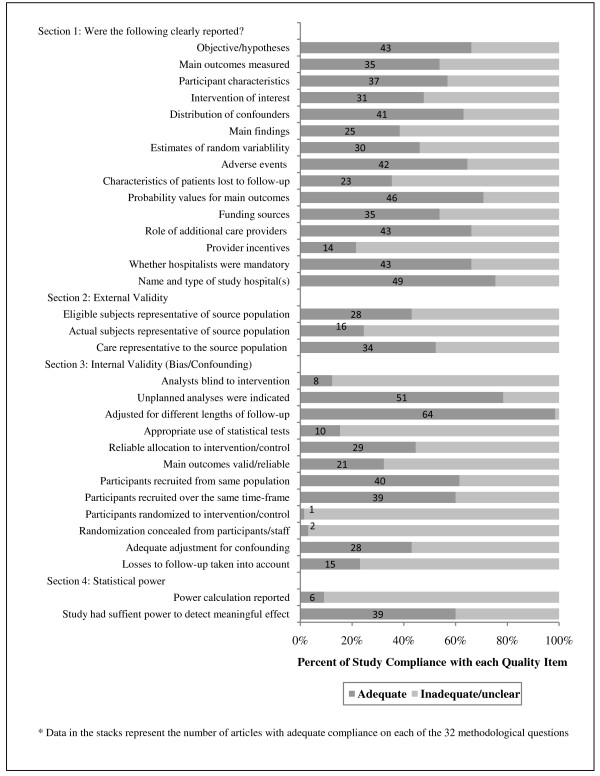
**Methodological critique of study reporting, validity, and statistical power (*n *= 65)**.

Despite more than a decade of research on hospitalist performance and several calls to improve the rigor of study design, reporting, and analyses [19,20,25,90], the methodological quality of comparative evaluations remains poor. The median quality score of the studies that we reviewed was 15 of a possible score of 32 (range, 5 to 26; see Tables 1 and 3), suggesting that more than half of all hospitalist evaluations published to date raise concerns regarding their reliability, validity, or transparency in reporting. The number and percentage of reviewed articles complying with each of the items included in our quality checklist are displayed in Figure 5. The quality of reporting and disclosure of information relevant to hospitalist interventions remain a concern in many publications; however, we highlight this as a promising area for methodological improvement. Thirty-four percent of all articles failed to state a clear objective of their evaluation in the introductory paragraphs (*n *= 22), and forty-three percent did not describe their sample and/or source populations or state patient inclusion or exclusion criteria (*n *= 30). More than half of reviewed articles did not describe the intervention and comparative care in enough detail to determine how many physicians actually delivered care to the patient sample and how this might have differed from care provided to the source population (*n *= 34). Only 22% of study authors included a statement on whether physicians were provided incentives to enhance their quality or efficiency (*n *= 14), and 34% of study authors did not indicate whether hospitalist care was mandatory for their patients (*n *= 22). These two issues are of particular concern, as their disclosure is necessary for interpreting the validity of any performance variations demonstrated across providers.

More than half of all studies also contained serious methodological errors, many of which could have been easily corrected. Twenty-six evaluations (40%) used insufficient sample sizes to demonstrate a clinically meaningful effect, and thirty-five (54%) appeared to use the wrong denominators when calculating incidence and risk for treatment outcomes (that is, readmission or follow-up rates calculated among all admissions as opposed to those who survived until discharge). Fifteen studies (23%) made no attempt to adjust findings for potential confounding or bias, and another twenty-two studies (34%) used partially adjusted models that excluded one or more known confounders, such as patient age, sex, and/or insurance status; case mix; and the severity of the patient's condition. Finally, while 51% of studies used analyses that adjusted for some confounding factors in multivariable models (*n *= 30), only 15% used the appropriate hierarchical or clustered methods necessary for linking physician characteristics to patient outcomes in studies of provider performance. All of these issues decrease the internal validity of hospitalist evaluations, making it difficult for readers, clinicians, and policy analysts to assess the extent to which improvements in performance outcomes can be attributed to hospitalist care as opposed to unmeasured or unadjusted confounding variables.

Restricting studies to those conducted since the publication of Coffman and Rundall's systematic review [19] demonstrated no improvement in methodological quality over time (*n *= 33; median quality score = 14; range, 5 to 22). Calculating each article's percentage rating (study score ÷ 32), articles with poor quality ratings (between 0% and 49%; *n *= 35) typically had missing descriptions of source populations, inclusion and exclusion criteria, and the number of hospitalist and comparative care providers, all of which limit the external validity and representativeness of potentially important findings. The majority of poor quality studies also failed to disclose numerators or denominators for their outcome data (78%) and estimates of random variability (75%) for one or more main indicators, making it impossible for readers to assess the accuracy of the authors' analyses and conclusions. In contrast, articles with high quality ratings (>70%; *n *= 6) were transparent in their reporting, used randomized or quasi-randomized designs (67%), and made extensive attempts to account for selection bias and known sources of confounding (100%), all of which translated to high internal and external validity. It should be noted that because of the nature of inpatient care, it would be difficult for any study evaluation to obtain a perfect quality score, as concealment of allocation, even after randomization, is rarely feasible.

To assess the sensitivity of our conclusions to the methodological quality of the literature, we examined performance outcomes for those studies that received adequate or high quality ratings (percentage rating ≥60%; *n *= 14). The findings appear to be consistent with our earlier conclusions, suggesting improved efficiency by hospitalist providers (86%) with no subsequent improvements in processes (67%) or clinical outcomes of care (71%). Of the three studies which showed better [49,67] or worse [80] performance by hospitalists in these areas, two of the findings were from unadjusted analyses [49,80]. In contrast, while poor quality articles (*n *= 35; 77%) were equally likely to report gains in efficiency, they were also more likely to report improvements in the processes (54%) and outcomes of care (30%), the majority of which were unadjusted (70%) and likely driven by confounding.

## Discussion

In this systematic review, we assessed the relationship between hospitalist physicians and the quality of inpatient care delivery. Forty-six of the sixty-five reviewed articles demonstrated that hospitalists delivered a higher quality of care to their patients compared to traditional inpatient physicians, and only seven studies indicated worse quality under the care of hospitalists. Superior outcomes were demonstrated across all care settings, regardless of study design, hospital type, patient eligibility, or physician practice structures. Stratifying these findings according to the area of quality examined showed improvements in operating efficiency among hospitalists (43 of 59 evaluations); however, there were few significant differences between physicians on process measures (15 of 26 evaluations) or clinical outcomes (33 of 52 evaluations). Taken together, our review of the current evidence suggests that hospitalists provide a level of clinical care that is comparable to that of their colleagues; however, their enhanced on-site availability and additional time spent on service suggests that the hospitalists' primary value likely comes from their ability to provide the same quality of clinical care in shorter periods of time, as evidenced by reductions in patients' average length of hospital stay reported in selected studies. Decreases in operating costs appear to be achieved largely by an increase in patient processing as opposed to reductions in the type and intensity of services provided. While there is no evidence to suggest that hospitalists provide a higher quality of clinical care, improvements in efficiency do not appear to come at the expense of clinical outcomes or patient and family satisfaction.

Despite these promising findings, many of the included studies had important methodological limitations, which decreases our confidence that findings reflect an accurate indication of hospitalist performance. Small sample sizes and inadequate statistical power were an issue in many studies, making it difficult to comment on whether hospitalists can decrease the incidence of rare outcomes such as in-hospital mortality or readmissions. The nonrandom allocation of patients frequently resulted in selection bias to preferred physician structures, where important covariates such as patient age, sex, ethnicity, insurance status, and preexisting comorbidities were often excluded from statistical models. Together, these factors resulted in poorly matched comparison groups and unadjusted biases. Finally, the statistical analyses used in selected studies were rarely conducted appropriately. Clinical indicators were frequently estimated among populations that were not actually at risk for the outcomes of interest, and inferences about quality were made at the level of providers without accounting for the clustering of patients within physicians. Furthermore, these methodological issues persist despite numerous calls urging researchers to enhance the rigor and reporting of the care provided by hospitalists compared with that offered by other healthcare providers.

Our findings are consistent with those reported in previous systematic reviews by Coffman and Rundall [19] and Landrigan *et al. *[17] suggesting improved performance by hospitalists based on the indicators of operating efficiency with no significant differences in patient outcomes between providers. These findings stand in contrast to those of Peterson's recent review [15], which found improvements in some process and outcome measures in addition to efficiency gains. It is worth noting that articles judged to be of 'poor' quality were excluded from Peterson's review, which may explain some of the deviations in our conclusions. When we attempted to replicate a version of Peterson's approach by excluding articles with quality scores below 50% (*n *= 35), we found little evidence to support processes or outcome improvements by hospitalists; however, 40% (*n *= 13) of the evaluations included in Peterson's review were found to have low quality scores in our review using the modified Downs and Black checklist [42].

In this systematic review, we propose a modified version of Donabedian's [26] framework as a simple conceptual map for understanding and synthesizing hospitalist performance, recognizing that an organization's structures, processes, and outcomes are interrelated and influence one another. By organizing these relationships into categories, researchers can logically predict and test relationships between constructs of interest and, in doing so, facilitate progression in the field of hospital medicine and quality initiatives. Structural differences between physician models should correlate with changes in the processes of care delivery, which in turn help drive improvements in operating efficiency and clinical outcomes. The results summarized in this review are important, as they suggest that the identification, labelling, and comparing of physicians as either 'hospitalists', 'traditional academic attending physicians', or 'community-based' providers is not sensitive enough to adequately differentiate the key structural characteristics which define hospitalists as distinct from other inpatient physicians and subsequently drive improvements in patient-level outcomes. The list of structural characteristics included in our conceptual model (Figure 1) quickly makes it apparent that inpatient physicians have access to many of the same resources and supports, regardless of job title, training, or time spent on service. By restricting all organizational aspects of a practice model to a single explanatory dummy variable as the vast majority of hospitalist evaluations have done, we do see evidence of improved performance in operating efficiency; however, we do not have a clear picture of where or how these efficiency gains occur and why we do not see similar improvement in related areas of quality (mainly processes and outcome measures).

Recognizing that hospitalists are now firmly entrenched within a large proportion of North American hospitals, if we wish to improve the quality of inpatient care delivery and introduce funding models that reward providers and/or institutions on the basis of their performance, further descriptive research labelling, categorizing, and analyzing of physicians according to their practice structures alone is unlikely to advance the research field in a way that will help inform organizational decision-making or health policy. Future research should instead shift toward developing better conceptual and theoretical models that identify and measure specific structural differences between physician practices, organizational issues that affect hospitalist groups, and the process mechanisms whereby hospitalist-based physicians have an increased opportunity to intervene.

On the basis of the findings of this review, we suggest that one of the key structural characteristics driving efficiency improvements among hospitalists is likely the increased time spent attending on the inpatient service and its subsequent impact on inpatient volume. Hundreds of articles published over the past three decades have shown that processes utilized and outcomes of care achieved are better among healthcare providers who perform them more frequently [6,91]. These volume-outcome associations have been demonstrated across a wide range of study designs, patient populations, health delivery models, and outcomes examined, and they persist despite extensive adjustment for organizational differences between institutions. While the categorical classification of hospitalists implies a volume-outcome relationship, only three studies included in this review specifically examined case volume at the provider level as an explanatory variable of quality outcomes [53,54,92]. Many hospitalists choose to practice part-time. As such, the annual volume and experience of a part-time hospitalist may actually approach that of some comparative providers, potentially washing out any improvements in quality that may be driven by volume as opposed to the portion of a physician's practice which is dedicated to inpatient care delivery, a common approach used to define hospitalists. This effect was demonstrated by Lindenauer *et al. *[92], who found that hospital length of stay and costs varied by <0.10 days and $15, respectively, among providers in models that were not adjusted for physicians' annual case volume.

By examining the quality of general inpatient care as a function of a physician's annual case volume, we can also extend the application of this literature to other healthcare models around the world which have instituted parallel inpatient practices without necessarily establishing formalized hospitalist programs. For example, inpatient care delivery in Australia, New Zealand, the United Kingdom, Singapore, and several other former British colonies is similar to the North American hospitalist model in that primary care is handed over to a separate system of specialists and consultants (most often general internists and/or general surgeons) once a patient is admitted. Like the hospitalist, the specialist then 'owns' the patient for the duration of hospitalization, providing the majority of their clinical services within the hospital setting. In this manner, several structural characteristics of the hospitalist and specialist models overlap: Both have high annual inpatient volume, which theoretically enhances clinical expertise and improves patient outcomes, and both operate in a routine environment where familiarity with staff, services, and technological resources support efficient practice. There are, however, a few key differences. Hospitalists tend to practice using a team-based approach where patients, call hours, and vacation time are rotated according to prearranged contracts, while specialists still tend to operate individually, negotiating their work hours directly with the hospital administration. Furthermore, inpatient specialists frequently hold higher levels of medical certification than many North American hospitalists, especially in Canada, where more than 90% of hospitalists hold only a general medical licence and no formalized training in hospital medicine [22]. Finally, there is the issue of incentives. Financial and other incentives for improving quality and efficiency are more common for hospitalists in the United States, while inpatient care in other countries is traditionally publicly funded. As a result, the need for these providers to modify their performance is frequently generated by negative pressure to reduce inefficiencies, potentially offsetting any intrinsic motivation to provide better care.

Interestingly, none of the hospitalist evaluations published to date have examined process indicators relating to the timeliness of care delivery, which would theoretically drive efficiency gains within our conceptual framework. In addition, transitions of care and communication patterns among hospitalists, patients, and their primary care physicians remain virtually unexplored and are important areas for further work. While computationally complex, this review highlights the need for multilevel, multisite studies which integrate the organizational effects of hospitals with more complete and informative data on the structure of hospitalist programs when undertaking evaluations of provider performance. Superior statistical models need to be used that control for patient, physician, and hospital-level confounding to understand whether higher inpatient quality reflects better hospital staffing and/or administration, organizational cultures that support hospitalist groups, or true improvements in the processes of care delivery by hospitalist physicians. Finally, the general quality of reporting in published studies can be improved by stating source populations, any inclusion versus exclusion criteria, patient and physician sample sizes within each comparison arm, and the number of patients lost to follow-up or excluded because of missing data. Disclosure of any performance incentives and funding sources, as well as the role of additional healthcare providers, should also be encouraged.

### Strengths and weaknesses

To our knowledge, this is the most comprehensive review of hospitalist performance conducted to date. While formal registration of the review was not undertaken, extensive attempts were made to prevent review-level bias, and the design, population, research questions, and literature search methods were all specified *a priori *according to the Participants, Interventions, Comparisons and Outcomes, or PICO, method [93] as well as the PRISMA guidelines [43]. We included studies of all methodological quality levels, with no restrictions on publication language, inpatient populations, physician practice structures, or outcomes examined. In addition, this is the first systematic review to assess the methodological quality of the hospitalist literature in which an objective checklist was employed that has been validated for use in both experimental and observational research [42]. We tested the sensitivity of our findings to methodological quality, demonstrating that our conclusions are supported in both high and low quality studies, but highlighted that poor quality studies were more likely to report better performance among hospitalists, a result which may have been driven largely by confounding. Finally, we have developed and presented a conceptual framework for synthesizing and evaluating hospitalist performance. By situating our conclusions within this underlying framework, we were able to identify several gaps in the evidence where hospitalist performance appeared to deviate from its theoretical foundation. We have highlighted key areas of interest that hospitalist researchers may wish to explore in the coming years.

Despite these strengths, several weaknesses in our review should be noted. Given the heterogeneity of designs and outcomes examined among studies, we were unable to conduct formal meta-analyses or generate summary estimates of risk for any of the outcome measures. While meta-analysis would be powerful for estimating the overall impact of hospitalists on the effectiveness and efficiency of inpatient care delivery, the validity of this approach rests largely on the quality of reporting in the original studies, and 53% of the reviewed studies did not report enough information to compute standard effect sizes and/or margins of error. The pooling of results is also considered inappropriate when unadjusted biases are suspected. Despite this limitation, decreases in the length of stay and the cost of care were demonstrated across all practice settings and patient populations, strongly suggesting that hospitalists do improve the efficiency of care delivery. Assessing the methodological quality of individual studies is widely accepted as good practice in systematic reviews of randomized, controlled trials; however, the use of quality assessment tools to appraise observational studies is less established. We used a validated and reliable checklist that has demonstrated high internal consistency and good test-retest and interrater reliability for both randomized and nonrandomized studies [42]. Nonetheless, each study is unique, and we recognize that a quality checklist may not include all items that are relevant for a particular topic and may include some items that are irrelevant, which can result in the misclassification of a study's quality. We attempted to minimize this risk by modifying the original Downs and Black checklist [42] to include several items specific to reporting within hospitalist comparisons and to remove one question that was not applicable to these designs. One author (HLW) extracted data from the selected publications which could introduce errors in our analyses; however, in those instances where required information was unclear, input was sought and consensus was reached between both authors. Finally, the majority of studies included in this review did not adjust for important confounders of quality such as patient age, sex, insurance status, comorbidities, and hospital and physician clustering. Recognizing that risk adjustment can have a profound impact on individual study results, any conclusions drawn from a systematic review of hospitalists' performance may change substantially, depending on the type of risk adjustment employed and on inclusion versus exclusion criteria. The trends identified in this review should be verified and reevaluated in the coming years as the methodological quality of new evaluations continues to improve.

## Conclusions

Despite the methodological limitations that decrease the quality of the published literature on hospitalist performance, common themes emerged from this review. Hospitalist physicians are efficient providers of inpatient care as observed by reductions in patients' average length of stay and total hospital costs; however, the clinical quality of hospitalist care is comparable to that provided by their colleagues. Opportunities for further research include an expanded focus on the specific structures of care that differentiate hospitalists from other inpatient physicians as well as on the development of better conceptual and statistical models that identify and measure the pathways of care that these structural differences are thought to influence.

## Competing interests

The authors declare that they have no competing interests.

## Authors' contributions

HLW conceived of the systematic review, performed data collection and drafted the manuscript for publication. RHG and HLW were jointly responsible for the study's design, analyses and interpretation. Both authors read and approved the final manuscript.

## Pre-publication history

The pre-publication history for this paper can be accessed here:

http://www.biomedcentral.com/1741-7015/9/58/prepub

## Supplementary Material

Additional file 1 **Individual study results on hospitalist performance. **Contains detailed results from the 65 included articles stratified by the type of quality area examined (Table 1: Processes of Care; Table 2: Operating Efficiency; Table 3: Clinical Outcomes). Click here for file

Additional file 2 **Checklist for assessing study quality, modified from Downs and Black**. [42]. Provides full details on our modified methodological checklist and all evaluation criteria used to assign quality ratings. Click here for file

Additional file 3 **PRISMA Checklist: Do Hospitalist Physicians Improve the Quality of Inpatient Care? **[43]. Click here for file
