# Supplementary material for: Do hospitalist physicians improve the quality of inpatient care delivery? A systematic review of process, efficiency and outcome measures
Source: BMC Med. 2011 May 18;9:58. doi: 10.1186/1741-7015-9-58 (PMC3123228; doi:10.1186/1741-7015-9-58)
Supplement: Additional file 1 — Individual study results on hospitalist performance. Contains detailed results from the 65 included articles stratified by the type of quality area examined (Table 1: Processes of Care; Table 2: Operating Efficiency; Table 3: Clinical Outcomes). [file 1741-7015-9-58-S1.DOC]

**Table 1.** Individual study results on hospitalist performance and process indicators of quality (n = 26)

| **Source** | **Type^1^** | **Sample^2^** | **Hospitalist Intervention** | **Comparison Group** | **Risk-Adjustment** | **Reported Results^3^** |
| --- | --- | --- | --- | --- | --- | --- |
| Abenhaim et al., (2000) | RC | N = 2722  I = 1094  C = 1628 | Faculty hospitalists  (n = 7) | Traditional academic attendings with house staff |  | Subspecialty consultations decreased by 42%*ǂ |
| Auerbach et al., (2002) | RC | N = 5308  I = 1615  C = 3693 | Academic hospitalist attendings (n = 5)  with house staff | Community-based physicians (n = 113)  with house staff | Regression: demographics, case-mix, clinical data, year | No difference in subspecialty consultations |
| Auerbach & Pantilat (2004) | RC | N = 148  I = 74  C = 74 | Academic hospitalist attendings (n = 5)  with house staff | Community-based physicians (n = 36)  with house staff | Regression: demographics, severity, clinical data, physician clustering | Hospitalists were 3.5 x more likely to have documented a family meeting and 4.8 x more likely to have prescribed a long-acting benzodiazepine in the 48 hours prior to death; no difference in the likelihood of providing skin or oral care |
| Bell et al., (2009) | QE | N = 1078  I = 371  C = 707 | Mixed practice types | Community-based  physicians & internal medicine subspecialists | Quasi-randomized based on physician's call cycle | No differences in communication patterns with outpatient physicians* |
| Bellet & Whitaker (2000) | B/A | N = 1440  I = 813  C = 627 | Pediatric hospitalist attendings (n = 10)  with house staff | Traditional academic attendings (n = 31) & community-based physicians (n = 13) |  | No difference in subspecialty consultations* |
| Davis et al., (2000) | RC | N = 2124  I = 211  C = 948 | Private hospitalists (n = 2)  assisted by a nurse manager | Community-based physicians (n = 17) |  | Hospitalists ordered 22%* fewer laboratory tests but 20%* more hematology services per patient; no differences in radiology*, EEG*, ECG*  or antibiotic utilization* |
| Dwight et al., (2004) | RC | N = 3807  I = 1274  C = 2533 | Faculty pediatric hospitalists (n = 3) | Traditional academic attendings with house staff | Regression: demographics, comorbidity | No difference in subspecialty consultations |
| Freese et al., (1999) | B/A |  | Private hospitalists  (n = 2) | Community-based physicians (n = 73) |  | Subspecialty consultations decreased by 17%*ǂ; no differences in laboratory*ǂ  or radiology*ǂ utilization |
| Go et al., (2010) | QE | N = 450  I = 177  C = 273 | Academic hospitalist attendings with house staff | Mixed practice types |  | No difference in ICU transfers* |
| Hackner et al., (2001) | PC | N = 1637  I = 477  C = 1160 | Academic hospitalist attendings (n = 10)  with house staff | Community-based physicians (n = 73) |  | Subspecialty consultations decreased  by 56%*; no differences in laboratory*, hematology*, OT/PT* utilization or  ICU transfers* |
| Kearns et al., (2001) | QE | N = 4455  I = 2238  C = 2217 | Academic hospitalist attendings (n = 4)  with house staff | Traditional academic attendings (n = 27)  with house staff | Randomization | No difference in ICU transfers* |
| Khasgiwali et al., (2006) | RC | N = 1916  I = 1173  C = 743 | Academic hospitalist attendings (n = 5) and private hospitalists (n = 3) | Traditional academic attendings (n = 82)  with house staff | Stratification by DRG | No differences in radiology utilization* |
| Lindenauer et al., (2002) | RC | N = 326  I = 137  C = 189 | Academic hospitalist attendings and private hospitalists (n = 20) | Community-based physicians (n = 65) | Regression: demographics | Hospitalists were 7% more likely to measure LVEF during admission; no differences in ACE-I/ARBs/ warfarin utilization or lifestyle counselling |
| Ogershok et al., (2001) | B/A | N = 2177  I = 1099  C = 1078 | Pediatric hospitalist attendings (n = 8)  with house staff | Traditional academic attendings with house staff |  | Hospitalists ordered 29% fewer laboratory*, 33% fewer hematology* and 20% fewer radiology* tests per patient |
| Palmer et al., (2001) | QE | N = 2464  I = 829  C = 1635 | Academic hospitalist attendings (n = 3)  with house staff and a nurse discharge planner | Traditional academic attendings (n = 27)  with house staff | Quasi-randomization based on physician's call cycle | Hospitalists ordered fewer laboratory*, hematology* and radiology* tests per patient compared to subspecialty attendings; no differences in the number of tests ordered between hospitalists and generalist attendings |
| Reddy et al., (2001) | RC | N = 151  I = 73  C = 78 | Academic hospitalist attendings with house staff | Mixed practice types | Regression: demographics, clinical data | No differences in laboratory, hematology or radiology utilization |
| Rifkin et al., (2002) | RC | N = 455  I = 185  C = 270 | Private hospitalists  (n = 9) | Community-based physicians (n = 56) | Stratification by severity | Mean time from stability to switch to oral antibiotics decreased by 35%*; no differences in appropriateness of initial antibiotics use* or the number of infectious disease/pulmonary consultations* |
| Rifkin et al., (2007) | RC | N = 158  I = 68  C = 90 | Faculty hospitalists  (n = 12) | Community-based physicians (n = 46) |  | Pts managed by hospitalists were 1.5 x more likely to receive DVT prophylaxis* and 1.3 x more likely to receive pneumococcal vaccination*; no differences in the % of pts receiving timely antibiotics*, blood cultures prior to antibiotic initiation* or smoking cessation counselling* |
| Roytman et al., (2008) | RC | N = 342  I = 126  C = 216 | Faculty hospitalists  (n = 15) | Community-based physicians | Regression: demographics, severity, comorbidity | Hospitalists were more likely to use ACE-I/ARBs within 24 hours of admission, prescribe IV diuretics and obtain social work consults but less likely to prescribe beta-blockers, obtain serial chest radiographs or multiple specialty consults; no differences in ECG use, PT/dietician consultation or sodium/fluid restrictions |
| Schneider et al., (2008) | QE | N = 1207  I = 495  C = 712 | Academic hospitalist attendings (n = 43)  with house staff | Traditional academic attendings (n = 171)  with house staff | Regression: demographics, hospital site, comorbidity, physician experience | No differences in pneumococcal vaccination, pain control or communication with outpatient physicians |
| Sharma et al., (2009) | RC | N = 21183 | Mixed practice types | Mixed practice types | Regression: demographics; comorbidity, hospital teaching status | Pts managed by hospitalists were 57% more likely to stay in the ICU during their final hospitalization |
| Smith et al., (2002) | RC | N = 45 I = 22  C = 23 | Private critical care hospitalists with house staff | Community-based physicians with house staff | Regression: demographics, severity, clinical data | Hospitalists were 4 x more likely to order serial chest radiographs; no differences in ICU transfers, antibiotic, hematology, laboratory utilization or palliative counselling |
| Somekh et al., (2008) | RC | N = 750  I = 250  C = 500 | Faculty hospitalist attendings (n = 8) | Community-based physicians and a cardiologist staffed chest-pain unit |  | Hospitalists ordered more stress MPIs*ǂ and 2-D echos*ǂ but fewer angiography* tests compared to community physicians; hospitalists ordered fewer stress MPIs* but more 2-D echos* and angiography tests*ǂ compared to cardiologists |
| Stein et al., (1998) | RC | N = 237  I = 114  C = 123 | Academic hospitalist attendings (n = 16)  with house staff | Community-based physicians (n = 52) with house staff or practicing solo (n = 39) |  | No differences in ICU transfers* |
| Vasilevskis et al., (2008) | RC | N = 372  I = 120  C = 252 | Mixed practice types | Mixed practice types | Regression: comorbidity, clinical data, hospital clustering | No differences in frequency of cardiac testing, LVEF measures or ACE-I/  ARB/beta-blocker prescribing |
| Wachter et al., (1998) | QE | N = 1623  I = 806  C = 817 | Academic hospitalist attendings (n = 14)  with house staff | Traditional academic attendings (n = 26)  with house staff | Quasi-randomization based on physician's call cycle | No difference in subspecialty consultation rates* |

^1^ Study designs include randomized control trials (RCT), quasi-experimental designs (QE) time-series (TS), prospective cohorts (PC), retrospective cohorts (RC),

before-after (B/A) and cross-sectional survey (CS)

**^2^** N = total sample size; I = hospitalist intervention same size; C = comparison sample size

^3^ * Indicates that results are based on unadjusted analyses; ǂ indicates that a p-value or confident interval was not provided - results may or may not be statistically significant

**Table 2.** Individual study results on hospitalist performance and efficiency indicators of quality (n = 59)

| **Source** | **Type^1^** | **Sample^2^** | **Hospitalist Intervention** | **Comparison Group** | **Risk-Adjustment** | **Reported Results^3^** |
| --- | --- | --- | --- | --- | --- | --- |
| Abenhaim et al., (2000) | RC | N = 2722  I = 1094  C = 1628 | Faculty hospitalists  (n = 7) | Traditional academic attendings with house staff |  | Median LOS decreased by 78%*ǂ  *(patients assigned to hospitalists based on brief anticipated LOS)* |
| Auerbach et al., (2002) | RC | N = 5308  I = 1615  C = 3693 | Academic hospitalist attendings (n = 5)  with house staff | Community-based physicians (n = 113)  with house staff | Regression: demographics, case-mix, clinical data, year | Median LOS decreased by 33%  Median costs reduced by 22% |
| Bekmezian et al., (2008) | RC | N = 925  I = 109  C = 816 | Faculty hospitalist (n = 1) | Traditional academic attendings with house staff | Regression-demographics, case-mix | Mean LOS decreased by 38%  Mean costs reduced by 29% |
| Bellet & Whitaker (2000) | B/A | N = 1440  I = 813  C = 627 | Pediatric hospitalist attendings (n = 10)  with house staff | Academic attendings  (n = 31) & community-based physicians (n = 13) | Regression: demographics, case-mix, physician characteristics | Mean LOS decreased by 11%  Mean costs reduced by 9% |
| Boyd et al., (2006) | RC | N = 1009  I = 740  C = 269 | Two private hospitalist teams (n = 4,5) both with house staff | Traditional academic attendings (n = 8)  with house staff | Regression: demographics, severity | Mean LOS increased by 12% - 19%  Mean costs increased by 10% |
| Carek et al., (2008) | RC | N = 5453  I = 1648  C = 3805 | Private hospitalists (n = 12) | Academic attendings with (n = 13) house staff and community-based physicians (n = 52) | Regression-demographics, severity (*hospitalists compared to teaching service only*) | Mean LOS increased by 18% compared to teaching service but decreased by 5%* compared to community physicians  Mean costs increased by 28% and 10%* respectively |
| Craig et al., (1999) | RC |  | Private hospitalist-staffed facilities | Non-hospitalist facilities | Demographics | Mean LOS decreased by 11% - 17%ǂ  Mean costs increased by 5% - 13%ǂ |
| Davis et al., (2000) | RC | N = 2124  I = 443  C = 1681 | Private hospitalists (n = 2)  assisted by a nurse manager | Community-based physicians (n = 17) | Demographics, case-mix | Mean LOS decreased by 25%  Mean costs reduced by 12% |
| Diamond et al., (1998) | B/A | N = 3299  I = 1620  C = 1679 | Academic hospitalist attendings with house staff | Community-based physicians with house staff |  | Mean LOS decreased by 27%*  Median costs reduced by 16%* |
| Dwight et al., (2004) | RC | N = 3807  I = 1274  C = 2533 | Faculty pediatric hospitalists (n = 3) | Traditional academic attendings with house staff | Regression: demographics, comorbidity | Mean LOS decreased by 14% |
| Dynan et al., (2009) | RC | N = 5543  I = 2383  C = 3160 | Faculty hospitalists (n = 8) assisted by a nurse practitioner | Traditional academic attendings (n = 40)  with house staff | Regression: demographics, case-mix, comorbidity | No difference in mean LOS  Mean costs reduced by 15% |
| Everett et al., (2004) | RC | N = 11750  I = 3133  C = 8617 | Private hospitalists (n = 27) | Community-based physicians (n = 131) | Regression: demographics, case-mix, year | Mean LOS decreased by 16%  Mean costs reduced by 8% |
| Everett et al., (2007) | RC | N = 22792  I = 11565  C = 11227 | Private hospitalists (n = 40) | Academic attendings  (n = 10) with house staff and community-based physicians (n = 52) | Regression: demographics, case-mix, severity, year | Mean LOS and costs increased by 42% and 32% respectively compared to academic attendings; mean LOS and costs decreased by 14% and 8% respectively compared to community-based physicians |
| Freese et al., (1999) | B/A |  | Private hospitalists  (n = 2) | Community-based physicians (n = 73) |  | Mean LOS decreased by 0.64 days*ǂ  Mean cost reduced by 25%*ǂ |
| Gittell et al., (2009) | RC | N = 6686 | Private hospitalists | Community-based physicians | Regression: demographics, severity, clinical data, physician clustering | Observed/expected LOS decreased by 36%; mean costs reduced by 6% |
| Go et al., (2010) | QE | N = 450  I = 164  C = 259 | Academic hospitalist attendings with house staff | Mixed practice types | Regression: demographics, severity, comorbidity, site, physician clustering | No difference in mean LOS  Mean costs reduced by 17% |
| Gregory et al., (2003) | B/A | N = 402  I = 93  C = 309 | Faculty hospitalist  (n = 1) | Traditional academic attendings with house staff |  | Mean LOS decreased by 37%*  Mean costs reduced by 24%* |
| Hackner et al., (2001) | PC | N = 1637  I = 477  C = 1160 | Academic hospitalist attendings (n = 10)  with house staff | Community-based physicians (n = 73) | Stratification by age and severity | Mean LOS and costs decreased by 16%* |
| Halasyamani et al., (2005) | RC | N = 10595  I = 6136  C = 4459 | Academic hospitalist attendings (n = 15) with house staff and private hospitalists (n = 18) | Community-based physicians (n = 63) | Regression: case-mix, physician clustering | Mean LOS and costs decreased by 20% and 10% respectively for academic hospitalists; mean LOS and costs decreased by 8% and 6% respectively for private hospitalists |
| Huddleston et al., (2004) | RCT | N = 469  I = 232  C = 237 | Faculty hospitalists (n = 3) comanaging with the orthopaedic team | Academic orthopaedic attendings (n =12)  with surgical residents | Randomization with adjustment for surgery type | Mean LOS decreased by 9%  No differences in mean costs |
| Kaboli et al., (2004) | QE | N = 1706  I = 447  C = 1259 | Academic hospitalist attendings (n = 3)  with house staff | Traditional academic attendings (n = 34)  with house staff | Regression: demographics, physician clustering | Mean LOS decreased by 16%  Mean costs reduced by 10% |
| Kearns et al., (2001) | QE | N = 4455  I = 2238  C = 2217 | Academic hospitalist attendings (n = 4)  with house staff | Traditional academic attendings (n = 27)  with house staff | Regression: demographics, diagnosis | No differences in mean LOS or costs |
| Khasgiwali et al., (2006) | RC | N = 1916  H = 1173  C = 743 | Academic hospitalist attendings (n = 5) and private hospitalists (n = 3) | Traditional academic attendings (n = 82)  with house staff | Stratification by DRG | No differences in mean LOS* or costs* |
| Krantz et al., (2005) | B/A | N = 493  I = 265  C = 228 | Private hospitalists (n = 6)  comanaging with cardiologists | Academic cardiologist attending with house staff |  | Median LOS decreased by 55%*  Time-to-admission decreased by 43%* |
| Kulaga et al., (2004) | RC | N = 2707  I = 583  C = 2124 | Academic hospitalist attendings (n = 2)  with house staff | Community-based physicians with house staff | Stratification by DRG | Mean LOS decreased by 21%*ǂ  Mean costs reduced by 18%ǂ* |
| Kuo et al., (2010) | RC | N=314590  I = 91065  C=223525 | Mixed practice types | Mixed practice types | Regression: demographics, case-mix, comorbidity, clinical data, hospital characteristics & clustering | Mean LOS decreased by 6% |
| Landrigan et al., (2002) | TS | N = 7748  I = 3625  C = 3823 | Academic hospitalist attendings with house staff and a nurse discharge planner | Community-based physicians | Time-series-temporal trend, case-mix | Mean LOS decreased by 12%  Mean costs reduced by 16% after the introduction of hospitalists - no concurrent improvements in LOS or cost among comparison HMOs |
| Lindenauer et al., (2002) | RC | N = 326  I = 137  C = 189 | Academic hospitalist attendings and private hospitalists  (n = 20) | Mixed practice types | Stratification by severity | Median LOS was equivalent or increased for pts with minor, moderate or severe illness and decreased for pts with major illness; no difference in median costs |
| Lindenauer et al., (2007) | RC | N = 76926  I = 24772  C = 52154 | Mixed practice types  (n = 284) | Mixed practice types  (n = 1964) | Regression: demographics, case-mix, physician volume, hospital characteristics; stratification by diagnosis | Mean LOS decreased by 12% compared to internists and family physicians;  Mean costs reduced by 5% compared to internists but not different from family physicians |
| Maa et al., (2007) | B/A |  | Academic surgical hospitalists (n = 3)  with house staff | Traditional surgical attendings with house staff |  | Time-to-surgery decreased by 50%* |
| Meltzer et al., (2002) | QE | N = 6511  I = 1613  C = 4898 | Academic hospitalist attendings (n = 2)  with house staff | Traditional academic attendings (n = 58)  with house staff | Regression: demographics, case-mix, comorbidity, physician clustering | No differences in LOS or costs in year one; mean LOS and costs decreased by 11% and 9% resp. in year two |
| Molinari & Short. (2001) | B/A | N = 1319  I = 903  C = 416 | Private hospitalists (n = 5) with nurse case manager | Community-based physicians (n = 59)  with nurse case manager | Regression: demographics | Observed/expected LOS was 74% more likely to fall within optimal guidelines |
| Ogershok et al., (2001) | B/A | N = 2177  I = 1099  C = 1078 | Academic pediatric hospitalist attendings  (n = 8) with house staff | Pediatric academic attendings with house staff |  | No difference in mean LOS*  Mean costs reduced by 13%* |
| Palacio et al., (2009) | RC | N = 5923  I = 3699  C = 2224 | Faculty hospitalists  (n = 14) | Traditional academic attendings (n = 8) with house staff |  | Mean LOS decreased by 16%* |
| Palmer et al., (2001) | QE | N = 2464  I = 829  C = 1635 | Academic hospitalist attendings (n = 3)  with house staff and a  nurse discharge planner | Traditional academic attendings (n = 27)  with house staff | Mixed effects ANOVA: demographics, case-mix, physician clustering  *(cost only*) | Mean LOS decreased by 17%* compared to generalist and 28%* compared to subspecialty attendings; mean costs reduced by 29% compared to subspecialty but not different for generalist attendings |
| Parekh et al., (2004) | RC | N = 2552  I = 913  C = 1639 | Academic hospitalist attendings (n = 7)  with house staff | Traditional academic attendings (n = 33)  with house staff | Regression: demographics, case-mix | No differences in mean LOS or costs |
| Phy et al., (2005) | B/A | N = 466  I = 230  C = 236 | Faculty hospitalists (n = 12)  comanaging with the orthopaedic team | Academic orthopaedic attendings with surgical residents | Regression: demographics, severity  *(time-to-surgery only*) | Mean LOS decreased by 21%*  Time-to-surgery reduced by 34% |
| Pinzuer et al., (2009) | B/A | N = 140  I = 86  C = 54 | Faculty hospitalists (n = 3)  comanaging with the orthopaedic team | Academic orthopaedic surgeon (n = 1) with  house staff | Regression-demographics, case-mix, comorbidity | Observed/expected LOS decreased by 20%ǂ; no difference in observed/expected costs |
| Ravikumar et al., (2010) | B/A | N = 9724  I = 1589  C = 3935 | Faculty hospitalists & physician assistants comanaging with the surgical team | Traditional surgical attendings with house staff |  | Mean LOS decreased by 16%* for patients admitted to the surgical ICU and 27% *for patients admitted to the progressive care unit |
| Reddy et al., (2001) | RC | N = 151  I = 73  C = 78 | Academic hospitalist attendings with house staff | Mixed practice types | Regression: demographics, case-mix | No differences in mean LOS or costs |
| Rifkin et al., (2002) | RC | N = 455  I = 185  C = 270 | Private hospitalists  (n = 9) | Community-based physicians (n = 56) | Regression: demographics, severity, clinical data | Mean LOS decreased by 14%  Mean costs reduced by 13% |
| Rifkin et al., (2004) | RC | N = 11388  I = 2027  C = 9361 | Faculty hospitalists  (n = 9) | Community-based physicians (n = 198) | Regression: demographics, case-mix, physician characteristics & clustering | No differences in the likelihood of having an above average LOS |
| Roy et al., (2006) | RC | N = 118  I = 47  C = 71 | Faculty hospitalists | Community-based physicians |  | No differences in median LOS* or costs*; % of pts receiving surgery within 24 hours of admission was 3 x higher* among hospitalists |
| Roytman et al., (2008) | RC | N = 342  I = 126  C = 216 | Faculty hospitalists  (n = 15) | Community-based physicians | ANCOVA: demographics, comorbidity; stratification by severity | Mean LOS decreased by 0-40%  Mean costs reduced by 14-28% |
| Salottolo et al., (2009) | B/A | N = 500  I = 261  C = 239 | Faculty hospitalists (n = 6) | Academic trauma physicians surgeons with house staff | Regression: demographics, clinical data | Mean LOS increased by 11% |
| Scheurer er al., (2005) | RC | N = 11969  I = 1214  C = 10755 | Mixed practice types  (n = 53) | Mixed practice types  (n = 1489) | Stratification by severity | Mean LOS decreased by 6-18%* for pts with moderate to severe illness but not different for pts with minor illness*; mean costs reduced by 10-26%* for pts with major and severe illness but not different for pts with minor/moderate illness |
| Schneider et al., (2008) | QE | N = 1207  I = 495  C = 712 | Academic hospitalist attendings (n = 43)  with house staff | Traditional academic attendings (n = 171)  with house staff | Regression: demographics, comorbidity, site,  physician experience | No differences in mean LOS or costs |
| Simon et al., (2007) | B/A | N = 759  I = 115  C = 644 | Faculty hospitalist (n = 1)  comanaging with the orthopaedic team | Academic orthopaedic team | Regression: demographics, clinical data, surgeon clustering | Mean LOS decreased by 26%  (only 12% *of post-intervention pts were actually co-managed by the hospitalist)* |
| Sloan et al., (2010) | B/A | N = 1409  I = 731  C = 679 | Faculty hospitalist  psychiatrists (n = 6)  with physician assistants | Psychiatrists providing continuity-of-care (n = 6) with physician assistants |  | No significant difference in mean LOS* |
| Smith et al., (2002) | RC | N = 45  I = 22  C = 23 | Private critical care  hospitalists with house staff | Community-based physicians with house staff | Regression: demographics, severity, clinical data | Mean LOS increased by 50%  Mean costs increased by 80% |
| Somekh et al., (2008) | RC | N = 750  I = 250  C = 500 | Faculty hospitalists (n = 8) | Community-based physicians and a cardiologist staffed  chest-pain unit | Regression: demographics, clinical data, comorbidity | Mean LOS increased by 11%ǂ compared community physicians and 278% compared to cardiologists |
| Southern et al., (2007) | RC | N = 9037  I = 2913  C = 6124 | Academic hospitalist attendings (n = 5)  with house staff | Traditional academic attendings with house staff | Regression: demographics, case-mix, clinical data | Mean LOS decreased by 22% |
| Srivastava et al., (2007) | B/A | N = 1970 | Pediatric hospitalist attendings (n = 3)  with house staff | Traditional academic attendings with house staff | Regression: demographics, severity | Mean LOS and costs decreased by 13% and 9% resp. for patients with asthma and by 11% and 8% resp. for patients with dehydration; no difference in mean LOS or costs for pts with viral illness |
| Stein et al., (1998) | RC | N = 237  I = 114  C = 123 | Academic hospitalist attendings (n = 16)  with house staff | Community-based physicians (n = 52) with house staff or practicing solo (n = 39) |  | Mean LOS and costs decreased by 21%* and 26% resp. compared to community-based physicians; mean LOS and costs decreased by 17%* compared to solo physicians with no differences in cost |
| Tenner et al., (2003) | B/A | N = 1211  I = 615  C = 596 | Private pediatric hospitalists (n = 5) | Pediatric intensivist attendings with house staff | Regression: severity, clinical data | Mean LOS decreased by 21 hours |
| Tingle and Lambert (2001) | RC | N = 529  I = 355  C = 174 | Faculty hospitalists  (n = 5) | Traditional academic attendings with house staff | ANOVA-severity | No differences in mean LOS or costs |
| Vasilevskis et al., (2008) | RC | N = 372  I = 120  C = 252 | Mixed practice types | Mixed practice types | Regression: comorbidity, clinical data, hospital clustering | No differences in mean LOS or costs |
| Wachter et al., (1998) | QE | N = 1623  I = 806  C = 817 | Academic hospitalist attendings (n = 14)  with house staff | Traditional academic attendings (n = 26)  with house staff | Regression: demographics, case-mix | Mean LOS decreased by 12%  Mean costs reduced by 10% |
| Wells et al., (2001) | PC | N = 181  I = 91  C = 90 | Private hospitalists (n = 5) | Community-based physicians (n = 37) | ANCOVA: demographics | Mean LOS and costs decreased by 32% and 44% resp. for pts with asthma; no differences in LOS or costs for pts with bronchitis, gastroenteritis or pneumonia |

^1^ Study designs include randomized control trials (RCT), quasi-experimental designs (QE) time-series (TS), prospective cohorts (PC), retrospective cohorts (RC),

before-after (B/A) and cross-sectional survey (CS)

**^2^** N = total sample size; I = hospitalist intervention same size; C = comparison sample size

^3^ * Indicates that results are based on unadjusted analyses; ǂ indicates that a p-value or confident interval was not provided - results may or may not be statistically significant

**Table 3.** Individual study results on hospitalist performance and clinical outcomes of treatment as indicators of quality (n = 51)

| **Source** | **Type^1^** | **Sample^2^** | **Hospitalist Intervention** | **Comparison Group** | **Risk-Adjustment** | **Reported Results^3^** |
| --- | --- | --- | --- | --- | --- | --- |
| Abenhaim et al., (2000) | RC | N = 272  I = 1094  C = 1628 | Faculty hospitalists  (n = 7) | Traditional academic attendings with house staff |  | In-hospital mortality decreased by 92%*ǂ; 30-day readmissions reduced by 31%*ǂ; Complications decreased by 84%*ǂ -(*Hospitalist* *patients selected based on brief anticipated LOS)* |
| Auerbach et al., (2002) | RC | N = 5308  I = 1615  C = 3693 | Academic hospitalist attendings (n = 5)  with house staff | Community-based  physicians (n = 113)  with house staff | Regression: demographics, case-mix, clinical data, year | Risk of in-hospital, 30-day & 60-day mortality decreased by 21-29%;  No differences in 10-day readmissions |
| Auerbach & Pantilat  (2004) | RC | N = 148  I = 74  C = 74 | Academic hospitalist attendings (n = 5)  with house staff | Community-based physicians (n = 36)  with house staff | Regression: demographics, severity, clinical data, physician clustering | Hospitalist pts were 2.7 x more likely to report being pain and anxiety free in the 48 hours prior to death |
| Batis et al., (2007) | B/A | N = 466  I = 230  C = 236 | Faculty hospitalists (n = 12)  comanaging with the orthopaedic team | Surgical orthopaedic or general teaching service |  | No difference in 1-year survival rates* |
| Bekmezian et al., (2008) | RC | N = 925  I = 109  C = 816 | Faculty hospitalist (n = 1) | Traditional academic attendings with house staff |  | No difference in in-hospital morality*;  72 hr readmissions were 4.4 x higher* |
| Bellet & Whitaker  (2000) | B/A | N = 1440  I = 813  C = 627 | Academic pediatric hospitalist attendings  (n = 10) with house staff | Academic attendings  (n = 31) & community-based physicians (n = 13) |  | 10-day readmissions were 3 x higher* among hospitalists |
| Carek et al., (2008) | RC | N = 5453  I = 1648  C = 3805 | Private hospitalists (n = 12) | Mixed academic attendings (n = 13) with house staff and community-based physicians (n = 52) | Regression: demographics, severity  (*hospitalists compared to teaching service only)* | No difference in 30-day readmissions |
| Craig et al., (1999) | RC |  | Private hospitalist-staffed facilities | Non-hospitalist facilities | Demographics | No difference in 7-day readmissionsǂ |
| Davis et al., (2000) | RC | N = 2124  I = 443  C = 1681 | Private hospitalists (n = 2) with a nurse manager | Community-based physicians (n = 17) | Stratification by DRG | No differences in in-hospital mortality*, 30-day readmissions* or pt satisfaction* |
| Dhuper & **Choksi** (2009) | B/A | N = 10966  I = 5508  C = 5458 | Academic hospitalist attendings (n = 12.5 FTEs) with physician assistants | Traditional academic attendings (n = 44.5 FTEs)  with house staff | Case-mix | In-hospital mortality reduced by 34%;  No differences in 30-day readmissions*, adverse events* or pt satisfaction* |
| Diamond et al., (1998) | B/A | N = 3299  I = 1620  C = 1679 | Academic hospitalist attendings with house staff | Community-based physicians with house staff |  | No differences in in-hospital mortality*; 14/30-day readmissions reduced by 54%* |
| Dwight et al., (2004) | RC | N = 3807  I = 1274  C = 2533 | Faculty pediatric hospitalists (n = 3) | Traditional academic attendings with house staff | Regression: demographics, comorbidity | No differences in in-hospital mortality or 7-day readmissions |
| Dynan et al., (2009) | RC | N = 5543  I = 2383  C = 3160 | Faculty hospitalists (n = 8) with a nurse practitioner | Traditional academic attendings (n = 40)  with house staff | Regression: demographics, case-mix | No differences in in-hospital mortality, 15-day or 30-day readmissions |
| Everett et al., (2004) | RC | N = 11750  I = 3133  C = 8617 | Private hospitalists (n = 27) | Community-based physicians (n = 131) | Regression: demographics, case-mix | No differences in in-hospital mortality or 30-day readmissions |
| Everett et al., (2007) | RC | N = 22792  I = 11565  C = 11227 | Private hospitalists (n = 40) | Mixed academic attendings (n = 10) with house staff and community-based physicians (n = 52) | Regression: demographics, case-mix | No differences in in-hospital mortality; 30-day readmissions reduced by 21% compared to academic attendings but not different from community-based physicians |
| Gittell et al., (2009) | RC | N = 6686 | Private hospitalists | Community-based physicians | Regression: demographics, severity, clinical data, physician clustering | No differences in in-hospital mortality or 7-day readmissions; 30-day readmissions reduced by 28% |
| Go et al., (2010) | QE | N = 450  I = 164  C = 259 | Academic hospitalist attendings with house staff | Academic attendings and community-based physicians, both with  house staff | Regression: demographics, severity, comorbidity, site, physician clustering (*complications only)* | No differences in in-hospital mortality*; 30-day readmissions* or complications |
| Gregory et al., (2003) | B/A | N = 402  I = 93  C = 309 | Non-academic hospitalist  (n = 1) | Traditional academic attendings with house staff |  | No difference in readmissions* |
| Hackner et al., (2001) | PC | N = 1637  I = 477  C = 1160 | Academic hospitalist attendings (n = 10)  with house staff | Community-based physicians (n = 73) |  | No differences in in-hospital mortality*, 14-day* or 30-day readmissions* |
| Halasyamani et al., (2005) | RC | N = 10595  I = 6136  C = 4459 | Academic hospitalist attendings (n = 15)  with house staff and private hospitalists (n = 18) | Community-based physicians (n = 63) | Regression: case-mix. physician clustering | No differences in in-hospital mortality, 30-day mortality or readmissions |
| Huddleston et al., (2004) | RCT | N = 469  I = 232  C = 237 | Faculty hospitalists (n = 3)  comanaging with the orthopaedic team | Academic orthopaedic attendings with surgical residents (n =12) | Randomization | Minor complications decreased by 32%; 24% more patients were discharged without any complications; no difference in pt satisfaction |
| Kaboli et al., (2004) | QE | N = 1706  I = 447  C = 1259 | Academic hospitalist attendings (n = 3)  with house staff | Traditional academic attendings (n = 34)  with house staff | Regression: demographics, physician clustering (*mortality only)* | No differences in in-hospital mortality or 30-day readmissions* |
| Kearns et al., (2001) | QE | N = 4455  I = 228  C = 2217 | Academic hospitalist attendings (n = 4)  with house staff | Traditional academic attendings (n = 27)  with house staff | Regression: demographics, diagnosis (*mortality only)* | No differences in in-hospital/30-day mortality or 7/30-day readmissions* |
| Khasgiwali et al., (2006) | RC | N = 1916  I = 1173  C = 743 | Academic hospitalist attendings (n = 5) and private hospitalists (n = 3) | Traditional academic attendings with house staff  (n = 82) |  | No difference in 30-day readmissions* |
| Krantz et al., (2005) | B/A | N = 493  I = 265  C = 228 | Private hospitalists (n = 6)  comanaging with cardiologists | Traditional cardiologist attending with house staff |  | No differences in 30-day readmissions* |
| Kulaga et al., (2004) | RC | N = 2707  I = 583  C = 2124 | Academic hospitalist attendings (n = 2)  with house staff | Community-based physicians with house staff |  | 30-day readmissions decreased by 32%* |
| Landrigan et al., (2002) | B/A | N = 7748  I = 3625  C = 3823 | Academic hospitalist attendings with house staff and a nurse discharge planner | Community-based physicians | Regression: demographics, severity | No differences in in-hospital mortality or 30-day readmissions |
| Lindenauer et al., (2002) | RC | N = 326  I = 137  C = 189 | Academic hospitalist attendings and private hospitalists (n = 20) | Community-based physicians (n = 65) |  | No differences in in-hospital mortality* or 30-day readmissions* |
| Lindenauer et al., (2007) | RC | N = 76926  I = 24772  C = 52154 | Mixed practice types  (n = 284) | Mixed practice types  (n = 1964) | Regression: demographics, case-mix, physician volume, hospital characteristics | No differences in in-hospital mortality or 30-day readmissions |
| Meltzer et al., (2002) | QE | N = 6511  I = 1613  C = 4898 | Academic hospitalist attendings (n = 2)  with house staff | Traditional academic attendings (n = 58)  with house staff | Regression: demographics, case-mix, comorbidity, physician clustering | 30-day mortality reduced by 35% in year two only; no differences in in-hospital/60-day/1-year mortality, 30-day readmissions, ED visits, self-reported health or pt. satisfaction |
| Ogershok et al., (2001) | B/A | N = 2177  I = 1099  C = 1078 | Academic pediatric hospitalist attendings  (n = 8) with house staff | Traditional pediatric academic attendings with house staff |  | No differences in in-hospital mortality*, 7-day* or 31-day* readmissions |
| Palacio et al., (2009) | RC | N = 5923  I = 3699  C = 2224 | Faculty hospitalists  (n = 14) | Traditional academic attendings (n = 8)  with house staff | Regression: demographics, clinical data | 30-day readmissions decreased by 26% |
| Palmer et al., (2001) | QE | N = 2464  I = 829  C = 1635 | Academic hospitalist attendings (n = 3)  with house staff and a nurse discharge planner | Traditional academic attendings (n = 27)  with house staff | Regression: demographics, case-mix, | In-hospital mortality reduced by 56% compared to subspecialty attendings but different from generalist attendings; no differences in 30-day readmissions or pt satisfaction |
| Parekh et al., (2004) | RC | N = 2552  I = 913  C = 1639 | Academic hospitalist attendings (n = 7)  with house staff | Traditional academic attendings (n = 33)  with house staff | Regression: demographics, case-mix | No differences in in-hospital mortality, 14-day or 30-day readmissions |
| Phy et al., (2005) | B/A | N = 466  I = 230  C = 236 | Faculty hospitalists (n = 12)  comanaging with the orthopaedic team | Academic orthopaedic attendings with surgical residents |  | No differences in in-hospital mortality*, 30-day readmissions* or complications* |
| Pinzuer et al., (2009) | B/A | N = 140  I = 86  C = 54 | Faculty hospitalists (n = 3)  comanaging with the orthopaedic team | Academic orthopaedic surgeon (n = 1) with  house staff |  | Complications increased by 250%*ǂ;  No differences in pt satisfaction*ǂ |
| Ravikumar et al., (2010) | B/A | N = 39769  I = 22270 C = 17499 | Faculty hospitalists & physician assistants comanaging with the surgical team | Traditional surgical attendings with house staff |  | In-hospital mortality decreased by 25%* |
| Rifkin et al., (2002) | RC | N = 455  I = 185  C = 270 | Private hospitalists  (n = 9) | Community-based physicians (n = 56) |  | No difference in in-hospital mortality, 15-day or 30-day readmissions |
| Roytman et al., (2008) | RC | N = 342  I = 126  C = 216 | Faculty hospitalists  (n = 15) | Community-based physicians | Regression: demographics, severity, comorbidity | In-hospital mortality decreased for pts managed by hospitalists; no differences in rates of acute renal failure or readmission |
| Salottolo et al., (2009) | B/A | N = 500  I = 261  C = 239 | Faculty hospitalists (n = 6) | Academic trauma physicians & surgeons with house staff | Regression: demographics | No differences in in-hospital mortality or complications |
| Schneider et al., (2008) | QE | N = 1207  I = 495  C = 712 | Academic hospitalist attendings (n = 43)  with house staff | Traditional academic attendings (n = 171)  with house staff | Regression-demographics, comorbidity, hospital site,  physician experience | No differences in in-hospital mortality, 30-day readmission, ED visit rates, pt satisfaction or self-reported health |
| Sloan et al., (2010) | B/A | N = 1409  I = 731  C = 679 | Faculty hospitalist  Psychiatrists (n = 6) with physician assistants | Psychiatrists providing continuity-of-care (n = 6) with physician assistants |  | 30-day readmissions decreased by 40%*; 30-day follow-up for a mental health visit increased by 20%* |
| Smith et al., (2002) | RC | N = 45  I = 22  C = 23 | Private critical care hospitalists with  house staff | Community-based physicians with house staff |  | No differences in in-hospital mortality*, 7-day readmissions* or 30-day ED visits* |
| Somekh et al., (2008) | RC | N = 750  I = 250  C = 500 | Faculty hospitalists  (n = 8) | Community-based physicians and a cardiologist staffed chest-pain unit | Regression-demographics, clinical data, comorbidity | Readmissions were 4x higher compared to cardiologists; no significant difference in readmissions between hospitalists and community physicians |
| Southern et al., (2007) | RC | N = 9037  I = 2913  C = 6124 | Academic hospitalist attendings (n = 5)  with house staff | Traditional academic attendings with house staff | Regression-demographics, case-mix, clinical data | No differences in in-hospital/30-day mortality or 30-day readmissions |
| Stein et al., (1998) | RC | N = 237  I = 114  C = 123 | Academic hospitalist attendings (n = 16)  with house staff | Community-based physicians (n = 52)  with house staff or practicing solo (n = 39) |  | No differences in in-hospital mortality* or 30-day readmissions* |
| Tenner et al., (2003) | B/A | N = 1211  I = 615  C = 596 | Private pediatric hospitalists (n = 5) | Pediatric intensivist attendings with house staff | Regression-severity, clinical data | Pts managed by hospitalists were 2.8 x more likely to survive until discharge |
| Tingle and Lambert (2001) | RC | N = 529  I = 355  C = 174 | Non-academic hospitalists  (n = 5) | Traditional academic attendings with house staff |  | No difference in in-hospital mortality |
| Vasilevskis et al., (2008) | RC | N = 372  H = 120  C = 252 | Mixed practice types | Mixed practice types | Regression: comorbidity, clinical data, hospital clustering | No differences in 30-day mortality or readmissions; likelihood of follow-up within 30-days of discharge increased by 83% |
| Wachter et al., (1998) | QE | N = 1623  I = 806  C = 817 | Academic hospitalist attendings (n = 14)  with house staff | Traditional academic attendings (n = 26)  with house staff | Regression: demographics, case-mix | No differences in in-hospital/6-month mortality, 10-day readmissions or self-reported health |
| Wells et al., (2001) | PC | N = 181  I = 91  C = 90 | Private hospitalists (n = 5) | Community-based physicians (n = 37) |  | No differences in 1-year readmissions, ED visit rates or 30-day follow-up visits; parents thought hospitalists were more courteous and friendly |

^1^ Study designs include randomized control trials (RCT), quasi-experimental designs (QE) time-series (TS), prospective cohorts (PC), retrospective cohorts (RC),

before-after (B/A) and cross-sectional survey (CS)

**^2^** N = total sample size; I = hospitalist intervention same size; C = comparison sample size

^3^ * Indicates that results are based on unadjusted analyses; ǂ indicates that a p-value or confident interval was not provided - results may or may not be statistically significant
