# Supplementary material for: Do hospitalist physicians improve the quality of inpatient care delivery? A systematic review of process, efficiency and outcome measures
Source: BMC Med. 2011 May 18;9:58. doi: 10.1186/1741-7015-9-58 (PMC3123228; doi:10.1186/1741-7015-9-58)
Supplement: Additional file 2 — Checklist for assessing study quality, modified from Downs and Black. [42]. Provides full details on our modified methodological checklist and all evaluation criteria used to assign quality ratings. [file 1741-7015-9-58-S2.PDF]

Additional File 2: Checklist for assessing study quality, modified from Downs & Black (1998).

Section 1: Reporting

1. *Is the objective/aim or hypotheses of the study clearly described within the introductory body of the manuscript?.*
If the objective is described only in the abstract, or not until the methods section, the question should be answered no.

Answer	Score
Yes	1
No	0

2. *Are the main outcomes to be measured clearly described in the Introduction or Methods section?*
If the main outcomes are first mentioned in the results section or aren't described in adequate detail for the reader to assess what was done, the question should be answered no.

Answer	Score
Yes	1
No	0

3. *Are the characteristics of the patients included in the study and the source population clearly described?*
Inclusion and/or exclusion criteria should be stated. Overall sample size must be stated.

Answer	Score
Yes	1
No	0

4. *Are the interventions of interest clearly described?*
Both hospitalist and control care should be clearly described. The number of physicians or FTEs providing care in each group must be stated.

Answer	Score
Yes	1
No	0

5. *Are the distributions of potential confounders in each group of subjects to be compared clearly described?*
A list of principle confounders (either descriptive in tabular format or in text within the methods or results section) is provided.

Answer	Score
Yes	1
No	0

6. *Are all main findings of the study clearly described?*
Quantitative outcomes data (including the numerator and denominator) should be reported for all main findings so that the reader can check any analyses and conclusions.

Answer	Score
Yes	1
No	0

7. *Does the study provide estimates of the random variability in the data for all main outcomes?*
In normally distributed data, the standard error, standard deviation or confidence intervals should be reported. In non-normally distributed data, the inter-quartile range should be reported. If the data distribution is not described, it must be assumed that the estimates used were appropriate and the question should be answered yes. If variances were provided for some but not all of the main outcomes, the question should be answered no.

Answer	Score
Yes	1
No	0

8. *Have all important adverse events that may be a consequence to the intervention been reported?*
This should be answered yes if the study demonstrated that there was a comprehensive attempt to measure and described significant adverse events related to inpatient care (i:e: in-hospital mortality, readmissions).

Answer	Score
Yes	1
No	0

9. *Have the characteristics of patients lost to follow-up been described?*
This should be answered yes when there were no losses to follow-up or when losses to follow-up were small and would not have affected the results by their exclusion. This should be answered no where a study does not report the number of patients lost to follow-up or where the number or patients excluded due to missing data was not described (retrospective studies).

Answer	Score
Yes	1
No	0

10. *Have actual probability values been reported for all main outcomes (i.e.: $p = 0.02$ rather than $p < 0.05$), except when the probability value is less than 0.001?*
Where probability values are provided for some but not all of the main outcomes, the question should be answered no.

Answer	Score
Yes	1
No	0

11. *Did the authors disclose sources of funding (if any)?*

Answer	Score
Yes	1
No	0

12. *Did the authors comment on the role of additional providers in the provision of inpatient care?*
If the authors disclosed any information relating to nursing/house-staff coverage or the provision of care provision by any other provider, the question should be answered yes.

Answer	Score
Yes	1
No	0

13. *Did the authors include a statement on whether incentives (monetary or otherwise) were provided to physicians to enhance their performance?*

Answer	Score
Yes	1
No	0

14. *Did the authors include a statement on whether the use of hospitalists were mandatory for managing the care of specific groups of inpatients?*

Answer	Score
Yes	1
No	0

15. *Did the authors disclose the name, geographic location and type of the hospital(s) where the study took place?*
For multisite evaluations, the source population and a description on how sites were selected must be included to answer yes. If only the type of hospital is disclosed, the question should be answered no.

Answer	Score
Yes	1
No	0

Section 2: External validity

16. *Were the subjects who were eligible to participate in the study representative of the entire population from which they were recruited?*
The study must identify the source population and describe how participants were selected in order to answer yes. Patients would be representative if they comprised the entire source population (i.e.: an entire year of hospitalizations to a general medical unit where study physicians comprise all practice structures for inpatient care), an unselected sample of consecutive patients, or a random sample. Where a study does not report the proportion of the source population from which the patients are derived or systematically excludes a portion of patients (i.e.: patients with private health insurance), the question should be answered no.

Answer	Score
Yes	1
No	0
Unable to determine	0

17. *Were subjects who actually participated representative of the entire population from which they were recruited?*
The proportion of patients who actually participated among those eligible to participate should be stated. Validation that the sample was representative would include demonstrating that the distribution of main confounders was the same in the study sample and source population.

Answer	Score
Yes	1
No	0
Unable to determine	0

18. *Were the staff and facilities where patients were treated representative of treatment the majority of patients would receive?*
For this question to be answered yes, the study should demonstrate that care in the study groups were similar to that which would be provided to the source population. The question should be answered no if for example, a large proportion of care is provided by a physician practice structures not included in the one of the study groups.

Answer	Score
Yes	1
No	0
Unable to determine	0

Section 3: Internal Validity-Bias

19. *Was an attempt made to blind those measuring the main outcomes to the intervention allocation?*

Answer	Score
Yes	1
No	0
Unable to determine	0

20. *If any of the results were based on "data dredging", was this made clear?*
Any analyses that had not been planned at the outset of the study should be clearly indicated and justified. If no unplanned analyses were reported, the question should be answered yes.

Answer	Score
Yes	1
No	0
Unable to determine	0

21. *Is the length of follow-up between the intervention and the outcome the same for all patients?*
Where follow-up is the same for all participants, the question should be answered yes. If different lengths of follow-up were adjusted for (i.e.: survival analyses), the question should be answered yes.

Answer	Score
Yes	1
No	0
Unable to determine	0

22. *Were the statistical tests used to assess the main outcomes appropriate?*
Statistical tests must be appropriate to the distribution of the data. Non-parametric methods should be used for non-normal data and small sample sizes. All data should be adjusted for the clustering of patients within physicians.

Answer	Score
Yes	1
No	0
Unable to determine	0

23. *Was compliance with the intervention appropriate?*
Where there was non-complication with treatment allocation of where there was significant contamination of one group by the other's care providers, the question should be answered no.

Answer	Score
Yes	1
No	0
Unable to determine	0

24. *Were the main outcomes used valid and reliable?*
For studies where the outcome measures are clearly described, the question should be answered yes. Denominators for outcome measures should include only the population at risk (for example, readmissions should only be counted among patients surviving to discharge).

Answer	Score
Yes	1
No	0
Unable to determine	0

Section 4: Internal Validity-Confounding/Selection Bias

25. *Were patients recruited from the same population?*
The question should be answered as unable to determine where information concerning the source population is not described.

Answer	Score
Yes	1
No	0
Unable to determine	0

26. *Were study subjects recruited over the same periods of time?*
Studies which used a before-and-after design should be answered no. For a study which does not specify the time period over which patients were recruited, the study should be answered as unable to determine.

Answer	Score
Yes	1
No	0
Unable to determine	0

27. *Were study subjects randomized groups?*
Studies which state that subjects were randomized should be answered yes except where the method of randomization would not ensure random allocation. For example, alternate allocation would score a 0 because allocation is predictable.

Answer	Score
Yes	1
No	0
Unable to determine	0

28. *Was the randomized assignment concealed from both patients and health care providers until recruitment was irrevocable?*
All non-randomized studies should be answered no.

Answer	Score
Yes	1
No	0
Unable to determine	0

29. *Was there adequate adjustment for individual-level confounding in the analyses from which the main findings were drawn?*
This question should be answered no for trials where: the main analyses were based on analyses of actual treatment rather than the intention to treat; the distribution of patient confounders were not described; or the distribution of known confounders was different between treatment groups and not taken into account in the analyses. In non-randomized studies, if the effects of patient-level confounders were not investigated or confounding was demonstrated but not adjusted for in all main analyses, the question should be answered no.

Answer	Score
Yes	1
No	0
Unable to determine	0

30. *Were losses of patients to follow-up taken into account in the analyses?*
If the proportion of patients lost to follow-up was too small to affect the main findings, the question should be answered yes. If the number of patients lost to follow-up are not reported, the question should be answered as unable to determine.

Answer	Score
Yes	1
No	0
Unable to determine	0

Section 5: Statistical Power

31. *Did the manuscript included a power or sample size calculation?*
Where a power or sample size analyses was undertaken, the question should be answered yes, even where the actual power/sample size was insufficient.

Answer	Score
Yes	1
No	0

32. *Did the study have sufficient power to detect a clinically important effect where the probability value for a difference being due to chance is less than 5%?*

Answer	Score
Yes	1
No	0
Unable to determine	0
